# Time‐encoded pseudo‐continuous arterial spin labeling: Increasing SNR in ASL dynamic angiography

**DOI:** 10.1002/mrm.29491

**Published:** 2022-10-18

**Authors:** Joseph G. Woods, S. Sophie Schauman, Mark Chiew, Michael A. Chappell, Thomas W. Okell

**Affiliations:** ^1^ Wellcome Centre for Integrated Neuroimaging, FMRIB, Nuffield Department of Clinical Neuroscience University of Oxford Oxford United Kingdom; ^2^ Department of Radiology Stanford University Stanford California USA; ^3^ Mental Health and Clinical Neurosciences, School of Medicine University of Nottingham Nottingham United Kingdom; ^4^ Sir Peter Mansfield Imaging Centre, School of Medicine University of Nottingham Nottingham United Kingdom; ^5^ Nottingham Biomedical Research Centre Queen's Medical Centre, University of Nottingham Nottingham United Kingdom

**Keywords:** angiography, arterial spin labeling, Hadamard, PCASL, time‐encoded

## Abstract

**Purpose:**

Dynamic angiography using arterial spin labeling (ASL) can provide detailed hemodynamic information. However, the long time‐resolved readouts require small flip angles to preserve ASL signal for later timepoints, limiting SNR. By using time‐encoded ASL to generate temporal information, the readout can be shortened. Here, the SNR improvements from using larger flip angles, made possible by the shorter readout, are quantitatively investigated.

**Methods:**

The SNR of a conventional protocol with nine Look‐Locker readouts and a 4 × 3 time‐encoded protocol with three Look‐Locker readouts (giving nine matched timepoints) were compared using simulations and in vivo data. Both protocols were compared using readouts with constant flip angles (CFAs) and variable flip angles (VFAs), where the VFA scheme was designed to produce a consistent ASL signal across readouts. Optimization of the background suppression to minimize physiological noise across readouts was also explored.

**Results:**

The time‐encoded protocol increased in vivo SNR by 103% and 96% when using CFAs or VFAs, respectively. Use of VFAs improved SNR compared with CFAs by 25% and 21% for the conventional and time‐encoded protocols, respectively. The VFA scheme also removed signal discontinuities in the time‐encoded data. Preliminary data suggest that optimizing the background suppression could improve in vivo SNR by a further 16%.

**Conclusions:**

Time encoding can be used to generate additional temporal information in ASL angiography. This enables the use of larger flip angles, which can double the SNR compared with a non‐time‐encoded protocol. The shortened time‐encoded readout can also lead to improved background suppression, reducing physiological noise and further improving SNR.

## 1 INTRODUCTION

**FIGURE 1:**
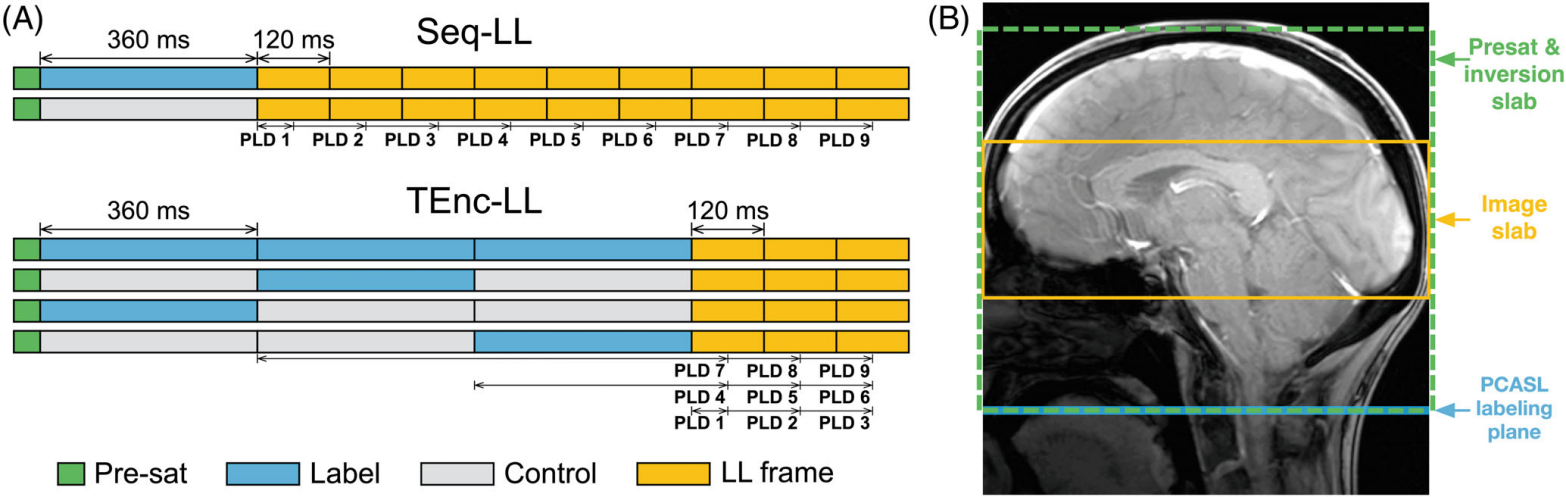
A,B, Outline of the protocol timings (A) and in vivo setup (B). A, The label and control ordering of the sequential Look‐Locker (Seq‐LL) and time‐encoded Look‐Locker (TEnc‐LL) protocols, demonstrating the equivalence of their decoded timings. The background suppression (BGS) inversion pulses are not shown for clarity. B, The positioning of the imaging slab, BGS (presaturation and inversion slabs), and the pseudo‐continuous arterial spin labeling (PCASL) labeling plane. Abbreviations: LL, Look‐Locker; PLD, postlabeling delay

Dynamic angiograms provide much richer hemodynamic^1^, ^2^ information than static angiograms and can be acquired with an arterial spin labeling (ASL) preparation and highly segmented time‐resolved Look‐Locker^3^ (LL) readouts.^4^, ^5^, ^6^ However, due to the large number of excitations, low flip angles are necessary to preserve signal for later timepoints, limiting SNR.^7^ By encoding some, or all, of the desired temporal information into the pseudo‐continuous ASL (PCASL) pulse train using a time‐encoded preparation,^8^ higher flip angles can be used because fewer excitations are required to achieve a given temporal resolution (see section 2 and Figure 1).^9^, ^10^, ^11^, ^12^ The resultant improvement in SNR could be used to balance increases to temporal or spatial resolution^1^, ^13^ which could help visualize small vessels implicated in lacunar infarction and vascular dementia, such as the lenticulostriate arteries.^14^ It may also help to improve the visibility of slow filling/draining arteriovenous malformations.^2^, ^13^, ^15^

While the SNR benefits of time‐encoded PCASL have been demonstrated for perfusion imaging,^16^, ^17^, ^18^ they have not yet been quantitatively investigated for ASL angiography.^9^, ^10^, ^12^, ^19^ The SNR in ASL‐based perfusion imaging is dependent on the accumulation of labeled spins in the tissue, so the shorter label durations (LDs) required with time‐encoded PCASL compared with sequential multi–postlabeling delay (PLD) PCASL can lead to a reduction in the overall SNR benefit of the technique for quantitative perfusion imaging.^18^ In contrast, ASL angiography is not dependent on tissue signal accumulation, because labeled blood is visualized as it traverses the arterial vasculature, meaning that short LDs do not decrease the peak signal (unless the LDs approach the timescale of bolus dispersion).

Here,^11^ we investigated the SNR benefits from using time‐encoded PCASL to generate some of the temporal information for dynamic angiography, enabling larger flip angles to be used during a LL spoiled gradient‐echo (SPGR) readout (this combination being referred to as TEnc‐LL). We also demonstrate how a variable flip angle (VFA) scheme can further improve SNR and remove signal discontinuities between temporal data decoded from different time‐encoded blocks.^10^, ^19^ However, because time encoding increases the minimum scan time, we demonstrate that SNR benefits remain after undersampling the TEnc‐LL acquisition by a factor of 2 to match the minimum scan duration of a fully sampled conventional LL scan (sequential LL [Seq‐LL]). Finally, we explore how shortening the readout allows for better suppression of background tissue signal, thus reducing physiological noise and further improving SNR.

## 2 THEORY

Time encoding enables multiple LDs and PLDs to be encoded into the PCASL preparation without using a LL readout^8^ or requiring the PLD/LD to be sequentially varied across ASL preparations.^20^ To achieve this, the PCASL preparation is split up into multiple distinct time‐encoded blocks. The label and control conditions for each block are varied independently over multiple ASL preparations using a Hadamard encoding scheme so that when the control‐label difference is decoded for a particular block, the ASL signal contributions from the other blocks cancel out (see Figure 3 in van Osch et al^21^ for a detailed illustration). When using a 4 × 3 Hadamard encoding scheme with a single readout frame, three separate PLDs can be decoded. By pairing this encoding with three LL readout frames (TEnc‐LL), a total of nine separate PLDs can be decoded,^9^ as demonstrated in Figure 1. This is similar to the time‐encoded multi‐PLD hybrid protocol proposed for perfusion imaging^18^ but with a LL readout instead of using multiple PLDs. Compared with an acquisition in which all PLDs are directly acquired from the LL readout frames (Seq‐LL), the readout duration and number of excitations are reduced by a factor of 3.

Reducing the number of excitations with time encoding enables larger flip angles to be used throughout the LL readout.^9^, ^12^ However, when constant flip angles (CFAs) are used, there can be large signal discontinuities between timepoints decoded from different time‐encoded blocks. This is because the data decoded from each time‐encoded block from later excitations have been cumulatively attenuated by the preceding excitations, whereas the data decoded from the earliest excitations have been minimally attenuated. To alleviate this problem, a VFA scheme can be used such that the flip angle increases during the readout to exactly compensate for the attenuation of the preceding excitation pulses, resulting in a consistent ASL signal over time.^10^, ^22^ For a SPGR readout, where the *n*th excitation pulse attenuates the ASL signal by a factor of cos(θ(n)) and θ(n) is the *n*th flip angle, the formula for such a VFA scheme can be defined using a backward recursive formula for a given maximum flip angle at the last excitation:

(1) $$\theta \left(n\right)=\tan^{-1}\left(\sin\left(\theta \left(n+1\right)\right)\right),$$

where n∈[1,…,N−1] and *N* is the total number of excitations in the readout. This is similar to the formula given by Wang et al,^6^ except that T_1_ relaxation is not included here because, ignoring dispersion, this will be constant for PCASL signal reaching a given voxel, unlike for PASL. The acquired ASL signal using this equation is maximized when θ(N)=90°.

## 3 METHODS

### 3.1 Protocols

We compared a conventional PCASL protocol with nine LL readout frames (Seq‐LL) to a TEnc‐LL protocol which used a 4 × 3 Hadamard encoding with three LL readout frames, giving nine decoded timepoints. Segmented SPGR readouts were used, each frame consisting of 12 excitation pulses (total number of excitations for all readout frames was 108 for Seq‐LL vs 36 for TEnc‐LL). The SPGR TR was 10 ms, giving a readout frame duration (and therefore temporal resolution) of 120 ms. To match the decoded PLDs between the Seq‐LL and TEnc‐LL protocols, the PCASL LD was 360 ms. The first readout frame started immediately after labeling, giving effective timepoints (LD + PLD) of 422, 542, 662, 782, 902, 1022, 1142, 1262, and 1382 ms, including the 2‐ms spoiler at the end of PCASL labeling.

### 3.2 Flip‐angle optimization

To fairly compare the Seq‐LL and TEnc‐LL PCASL angiography protocols, we optimized the excitation flip angles for each protocol for two cases: CFAs and VFAs. To maximize the visibility of distal vessels, which are filled at later timepoints, the optimization maximized the minimum theoretical ASL signal across the readout, equivalent to maximizing the profit function:

(2) $$R\left(N\right)=\sin\left(\theta \left(N\right)\right)\cdot \prod_{n=1}^{N-1}\cos\left(\theta \left(n\right)\right),$$

where we assume a constant supply of labeled blood during the readout; all labeled blood experiences every excitation; and there is perfect spoiling of transverse magnetization between excitations. This criterion is equivalent to maximizing the signal at the last excitation for CFAs, meaning Equation 2 can be simplified to $R\left(N\right)=\sin\left(\theta \right)\cdot \cos^{N-1}\left(\theta \right)$. Solving $\frac{dR}{d\theta }=0$ for *θ* gives the optimal CFA in terms of *N* as

(3) $$\theta \left(N\right)=\pi -2\cdot \tan^{-1}\left(\sqrt{2\cdot N-1+2\cdot \sqrt{\left(N-1\right)\cdot N}}\right).$$

Equation 2 is maximized for the VFA formula in Equation 1 when θ(N)=90°. However, to reduce specific absorption rate and the effect of variable slice profiles^23^ during the readout, we set θ(N)=30°. This only leads to a 1% and 4% decrease in the mean signal during the Seq‐LL and TEnc‐LL readouts, respectively (see Supporting Information Figure S1).

### 3.3 Signal model simulations

To investigate the advantages of the TEnc‐LL protocol over the Seq‐LL protocol, and the VFA scheme over CFA, we used a realistic signal model of labeled blood passing through a single voxel. This model, introduced by Okell et al,^24^ takes into account the LD and PLDs and incorporates RF attenuation, T_1_ relaxation, and dispersion (characterized by a gamma dispersion kernel). For simplicity, we assume all labeled blood is attenuated by all excitations, which gives the following model for the acquired ASL signal:

(4) $$\begin{array}{c} S(t)=A\cdot R\left(N^{\prime }\right)\cdot \frac{s^{1+p\cdot s}}{\Gamma (1+p\cdot s)}\cdot \int_{t-\Delta t-\tau }^{t-\Delta t}e^{-s\cdot t^{\prime }}\cdot {\left(t^{\prime }\right)}^{p\cdot s}\cdot e^{-\frac{\Delta t+t^{\prime }}{T_{1}}}\cdot dt^{\prime } \end{array}$$

where Γ(x) is the gamma function evaluated at x; s is the dispersion kernel sharpness (units of s^−1^); and p is the kernel time‐to‐peak (units of s). We also have that $N^{\prime }=\left\lceil \frac{t-t_{0}}{\mathrm{TR}}\right\rceil$, where *t* is the time from the start of PCASL labeling, t_0_ is the time at the center of the first excitation pulse, Δ*t* is the bolus arrival time, *τ* is the LD, and TR is the repetition time of the readout (10 ms).

Two cases were considered: (1) no dispersion, with *s* = 10^4^ s^−1^ and *p* = 0 s, and (2) moderate dispersion, with *s* = 10 s^−1^ and *p* = 0.1 s. To aid the comparison with the in vivo data, the moderate dispersion case was down‐sampled from a 10‐ms temporal resolution to the temporal resolution of the in vivo reconstructed data (120 ms) by taking the mean signal across the 12 excitations in each readout frame.

### 3.4 In vivo comparisons

Six volunteers (3 female, mean age 31 ± 9 years) were recruited and scanned under a technical‐development protocol, under agreement with local ethics and institutional committees, on a 3T Verio system (Siemens Healthcare, Erlangen, Germany) with a 32‐channel receive‐only head coil. All scanning occurred during a single scan session for each volunteer. The volunteers were asked to lie still during the scan but were not required to stay awake.

The ASL data were acquired using a 2D‐SPGR radial readout, similar to that used in Berry et al,^25^ to facilitate rapid scans so that all of the protocols could be acquired within a single session. A three‐plane localizer was used to place the 70‐mm‐thick, transverse, ASL imaging slab approximately centered at the inferior edge of the thalamus with the superior edge including the corpus callosum (Figure 1B). A single‐slab 3D time‐of‐flight sequence (0.31 × 0.31 × 1.3 mm^3^) was used to place the transverse PCASL labeling plane at the middle of the V3 section of the vertebral arteries.

#### 3.4.1 Acquisition

Two different angiography spatial resolutions were used: (1) a lower in‐plane resolution of 1.15 × 1.15 mm^2^ to facilitate short scan times, enabling the SNR of all four protocols (Seq‐LL and TEnc‐LL with both CFA and VFA schemes) to be quantitatively compared; and (2) a higher in‐plane resolution of 0.63 × 0.63 mm^2^, to qualitatively compare the visibility of small distal vessels for the VFA protocols.

Common imaging parameters were FOV = 220 × 220 mm, TR = 10 ms, 12 excitations per readout frame, fully sampled at *k*_max_, temporal resolution = 120 ms, quadratic RF spoiling^26^ (50° increment), and no fat suppression. The radial spokes were acquired in sequential angular order within each readout frame, with the sequence acquiring the same spokes for each tag/control/encoding before looping over the next set of spokes until k‐space is filled at all timepoints. The spokes were angularly‐evenly distributed.

The lower‐resolution specific imaging parameters were as follows: matrix = 192 × 192, bandwidth = 131 Hz/pixel, TE = 5.08 ms, 300 radial spokes, 25 segments (ASL preparations per label/control/encoding), and 2:30 min scan time. The Seq‐LL protocols used two averages to match the scan time of the TEnc‐LL protocols (which require four encodings). These data were acquired in five subjects.

The higher‐resolution specific imaging parameters were as follows: matrix = 352 × 352, bandwidth = 138 Hz/px, TE = 5.27 ms, 552 radial spokes, 46 segments, and 4:35 min scan time. For this resolution, the Seq‐LL VFA protocol used 1104 separate radial views (92 segments) to match the scan time of the TEnc‐LL VFA protocol. This factor of two angular oversampling will result in similar noise averaging to the two‐average case, assuming no motion, and therefore represents an alternative method for collecting twice as much data for the Seq‐LL protocol. These data were acquired in four subjects.

Balanced PCASL labeling used 500‐μs Gaussian RF pulses, 1‐ms pulse interval, 20° flip angle, 0.8‐mT/m mean gradient, and 6‐mT/m selective gradient. Background suppression (BGS) used water suppression–enhanced–through–T_1_ effects presaturation^27^, ^28^ immediately before PCASL labeling and two C‐FOCI inversion pulses^29^, ^30^ (max $B_{1}^{+}\approx$ 10 μT due to specific absorption rate restrictions, *A*_max_ = 20, *μ* = 1.5 and *ß* = 1349.17 rad/s, and duration = 10.24 ms) interleaved with the PCASL labeling. The PCASL condition was switched from label to control and vice versa after each BGS inversion pulse to maintain the correct difference signal.^31^, ^32^ The inversion pulses were timed to null two T_1_ values (700 and 1400 ms) 100 ms before the first readout excitation using the equation given by Günther et al^33^:

(5) $$\begin{array}{cc} & t_{\mathrm{BGS}1}=\mathrm{TI}+2\cdot T_{1,\mathrm{opt}}\cdot \ln\left(\frac{1}{4}+\frac{3}{4}\cdot e^{-\frac{\mathrm{TI}}{2\cdot T_{1,\mathrm{opt}}}}\right)\\ & t_{\mathrm{BGS}2}=\mathrm{TI}+2\cdot T_{1,\mathrm{opt}}\cdot \ln\left(\frac{3}{4}+\frac{1}{4}\cdot e^{-\frac{\mathrm{TI}}{2\cdot T_{1,\mathrm{opt}}}}\right), \end{array}$$

where *t*_BGSn_ is measured from the start of labeling; TI = LD_total_ + PLD‐*t*_null_; LD_total_ = 360 ms for Seq‐LL and 1080 ms for TEnc‐LL; PLD = 2 ms; *t*_null_ = 100 ms; and T_1,opt_ = 700 ms.

#### 3.4.2 Readout undersampling

When using a 4 × 3 Hadamard encoding, the minimum scan time is twice that of the Seq‐LL protocol, because four encodings must be acquired rather than two (label and control). Therefore, to match the TEnc‐LL scan time, we acquired either two averages (low‐resolution) or twice as many spokes (high‐resolution) with the Seq‐LL protocol.

However, ASL angiograms are often acquired with only a single average in practice. To investigate the benefit of the TEnc‐LL VFA protocol with the scan time matched to that of one average of the Seq‐LL protocol, we prospectively angularly undersampled the readout of the low‐resolution TEnc‐LL VFA scan by a factor of ≈2 (13 segments compared with 25 for fully sampled), because the scan time is proportional to the number of segments. This scan (1:18 min) was acquired for all volunteers in the low‐resolution comparison and was compared with the first average of the low‐resolution Seq‐LL VFA scan (1:15 min).

#### 3.4.3 Optimized background suppression

For these experiments, the BGS inversion pulses were timed to null background tissue signal 100 ms before the first excitation, a common approach when using magnitude subtraction to avoid subtraction errors.^33^ This results in the static tissue signal increasing during the readout, with physiological noise correspondingly increasing. A more ideal approach when using complex subtraction, which we have used here (see section 3.4.4), would be to null the background tissue signal during the readout such that physiological noise is optimally reduced throughout the readout.

To explore this idea, we aimed to minimize the maximum absolute background tissue transverse magnetization during the readout as follows:

(6) $$\min_{t_{\text{null},\text{noRF}}}\left[\max\left(\left|M_{z}\cdot \sin\left(\theta \right)\right|\right)\right],$$

where *t*_null,noRF_ is identical to *t*_null_ in Equation 5 (the time when the two target tissue T_1_ values, 700 and 1400 ms, would be nulled relative to the first readout excitation) but is so named to make clear that it ignores the effect of the readout RF pulses (note, *t*_null,noRF_ < 0 corresponds to after the first excitation). This optimization approach means that we search over only a single parameter, *t*_null,noRF_, simplifying the implementation compared with directly optimizing the two BGS inversion timings. The value of **M**_**z**_ is an array of the longitudinal tissue magnetization just before the *n*th excitation, and **θ** is an array of the excitation flip angles, where

(7) $$M_{z}\left(n\right)=1-\left[1-M_{z}\left(n-1\right)\cdot \cos\left(\theta \left(n-1\right)\right)\right]\cdot e^{-\frac{\mathrm{TR}}{T_{1t}}}$$

for n∈[2,…,N], and

(8) $$M_{z}\left(1\right)=1-e^{-\frac{t_{\text{null},\text{noRF}}}{T_{1t}}},$$

where $t_{\text{null},\text{noRF}}>-T_{1t}\cdot \ln\left(2\right)$, TR = 10 ms, and *T*_1t_ is the longitudinal relaxation time of tissue, which we set to the average of white matter and gray matter^34^: (0.791s+1.445s)/2=1.118s. This simulation assumes instantaneous RF pulses and perfect spoiling.

The null time was optimized for the Seq‐LL and TEnc‐LL VFA protocols using a grid search between $-T_{1t}\cdot \ln\left(2\right)$ and 1 s, with a 1‐ms resolution. The inversion pulse timings were constrained to occur during the LD and not to overlap each other. To quantitatively evaluate the effect of this BGS timing optimization on the SNR, in vivo angiography data for both protocols were acquired in three of the five subjects in the low‐resolution comparison.

#### 3.4.4 Reconstruction

Offline image reconstruction was performed for the radial angiography data in *MATLAB* (2021a; The MathWorks, Natick, MA) as implemented in https://github.com/SophieSchau/Accelerated_TEASL.

Before reconstruction, the mean phase difference between matching spokes of the first acquired label/encoding and the following label/control/encoding conditions was subtracted to reduce B_0_ drift artifacts using $k_{m,\text{corrected}}=k_{m}\cdot e^{-i\cdot \phi }$, where $\phi =∠\left(k_{1}^{H}\cdot k_{m}\right)$ and $k_{m}$ is a single radial k‐space spoke across coil channels for the *m*th label/control/encoding condition. This phase correction assumes that static tissue, which should be identical in each image, is the dominant signal source and that any difference in phase is due to B_0_ drift. Supporting Information Figure S2 illustrates the reduction in subtraction artifacts after applying this bulk phase correction. While static tissue will not always be the dominant source of signal when the background is nulled during the readout, we did not notice adverse effects in these cases. This is likely because different tissue types are nulled at different times, so there is always a reasonable amount of static tissue signal to use as a phase reference. To further reduce the impact of B_0_ drift, the sequence acquired the same spokes for each label/control/encoding condition and any averages before acquiring the next set of spokes, thus minimizing the time between matched k‐space data.

After phase correction, density‐compensation weights were calculated using the fixed‐point method^35^ and applied. The adjoint of the nonuniform fast Fourier transform with min‐max interpolation^36^, ^37^ was used to perform a regridding reconstruction. Coil sensitivities were estimated using the adaptive combine algorithm^38^ with a kernel size of $\left\lceil \frac{10\;\mathrm{mm}}{\mathrm{res}}\right\rceil$ and a threshold of zero, where res is the in‐plane resolution. Roemer coil combination^39^ was used followed by complex subtraction/decoding. Finally, the magnitude operator was applied to the complex difference images.

#### 3.4.5 Signal‐to‐noise ratio quantification

**FIGURE 2:**
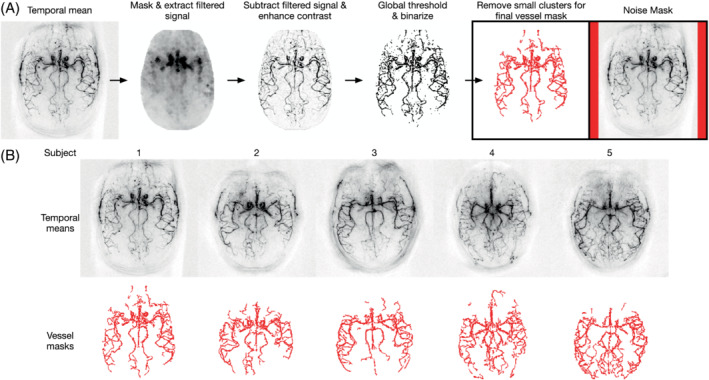
A, Automated process for creating the vessel masks for the low‐resolution comparison with the background noise mask shown in red on the right. B, The temporal mean images and vessel masks for each subject. The vessel mask used the following heuristic steps: (1) Rescale temporal mean to range [0,1]; (2) apply the brain mask and remove the filtered signal calculated using the *MATLAB* “strel” and “imopen” functions with a two‐voxel disk radius; (3) enhance contrast by saturating the top 1% of voxels; (4) apply a global threshold based on 50% of Otsu's threshold^40^ and binarize; and (5) remove clusters containing fewer than 10 voxels using the *MATLAB* “bwareaopen” function

A vessel mask was generated for each volunteer using the process outlined in Figure 2 from the combined temporal mean across the four scans in the fully sampled low‐resolution comparison. For this process, brain masks were manually drawn for each subject. To calculate the SNR, the mean signal within the vessel mask was divided by the noise SD within the background regions of interest (ROIs) at the edges of the image. Because the magnitude operator had been used, the noise SD was estimated as $\mathrm{RMS}/\sqrt{2}\;$ because this is equal to the underlying noise SD, assuming the complex background noise is normally distributed with zero mean and equal SD for the real and imaginary parts. Because of the use of coil sensitivities in the reconstruction, the noise magnitude will vary spatially across the image, but this will occur in a similar manner for all protocols. Significant differences between the calculated SNRs were compared on the subject level using two‐tailed paired t‐tests and the Holm‐Bonferroni correction for multiple comparisons, using *p* = .05.

## 4 RESULTS

### 4.1 Flip‐angle optimization

**FIGURE 3:**
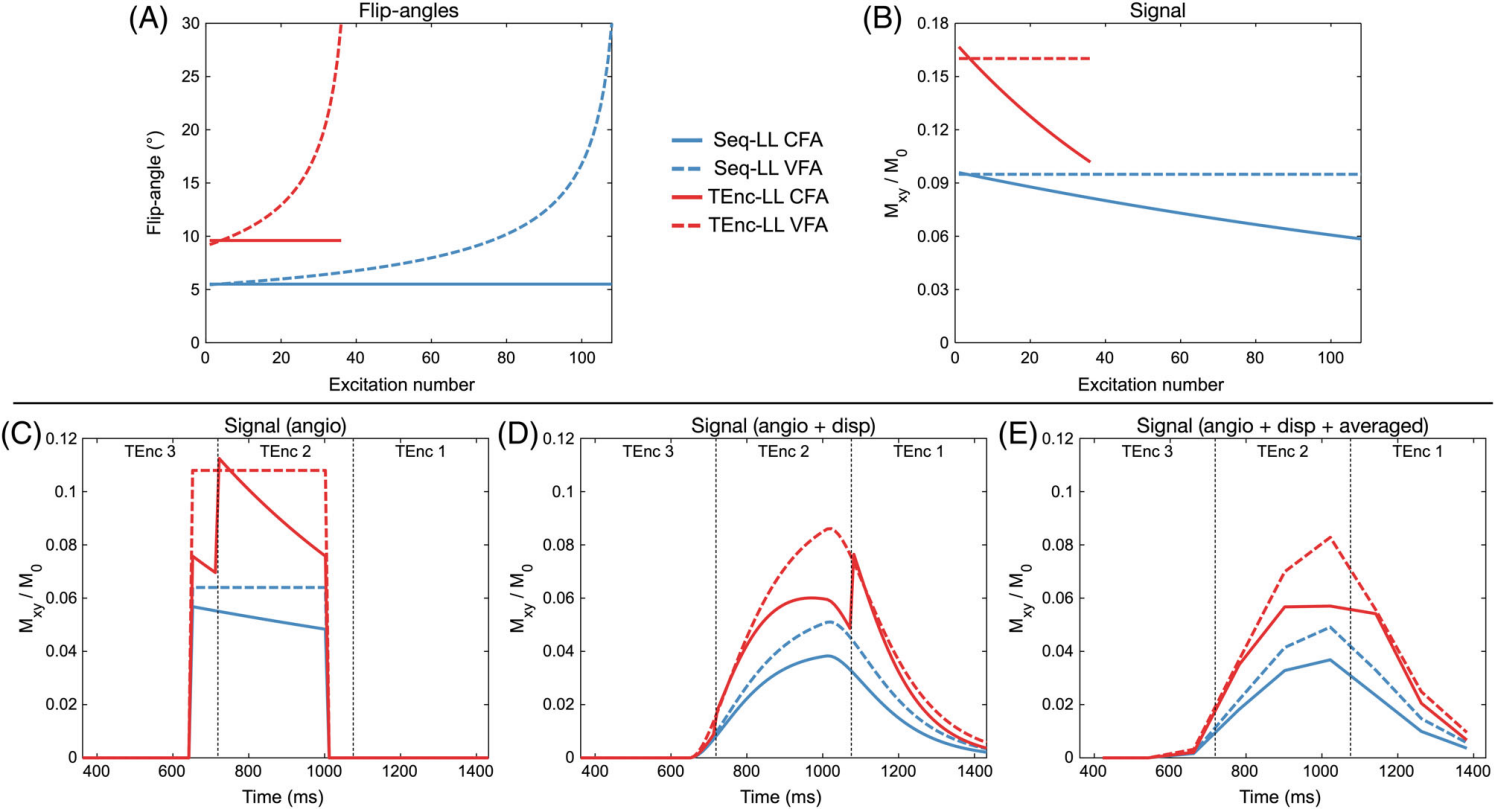
A, Optimized flip angles for the constant flip angle (CFA) and variable flip angle (VFA) schemes for the Seq‐LL and TEnc‐LL protocols. B, The simulated acquired arterial spin labeling (ASL) signal for each set of flip angles assuming a constant supply of ASL signal and zero arrival time. C–E, The simulated acquired ASL signal using a realistic ASL angiography signal model assuming no dispersion (*s* = 10^4^ s^−1^, *p* = 0 s) (C), moderate dispersion (*s* = 10 s^−1^, *p* = 0.1 s) (D), and moderate dispersion with temporal down‐sampling to match the in vivo data (E). For (C)–(E), the arrival time was 650 ms and *T*_1b_ = 1.65 s. “TEnc X” in (C)–(E) refers to the time‐encoded block from which the TEnc‐LL data are decoded

The CFA flip‐angle optimization resulted in the following flip angles: Seq‐LL = 5.5° and TEnc‐LL = 9.6°. The minimum flip angle for the VFA formula was Seq‐LL = 5.45° and TEnc‐LL = 9.21°; the maximum flip angle was 30° in both cases, as described previously. The flip angles are shown in Figure 3A.

The simulated signal for the constant supply signal model for each case is shown in Figure 3B. The shorter TEnc‐LL readout enabled higher signal to be acquired, whichever flip angle scheme was used (mean signal improvement relative to Seq‐LL: CFA = 74%, VFA = 69%). The VFA formula achieved its aim of acquiring constant signal during the readout. It also achieved a higher signal than CFA for most of the readout, due to the ramp in flip angles (mean signal improvement relative to CFA: Seq‐LL = 25%, TEnc‐LL = 22%).

### 4.2 Signal model simulations

The simulations using the realistic signal model are shown in Figure 3C–E. The signal gain from the larger flip angles possible with time encoding is also evident with this model. The use of CFAs with TEnc‐LL causes large signal discontinuities between timepoints decoded from different time‐encoded blocks, but these discontinuities are smoothed out when using VFAs. Although the signal discontinuities are less obvious at the lower temporal resolution (Figure 3E), the use of VFAs still leads to a clear signal gain compared with CFAs.

When dispersion was included and the temporal resolution matched the in vivo data (Figure 3E), the relative signal gains were as follows: TEnc‐LL versus Seq‐LL mean signal improvement CFA = 84% and VFA = 69%; and VFA versus CFA mean signal improvement Seq‐LL = 33% and TEnc‐LL = 22%. Note: The relative signal gains will depend on the exact model parameters used.

### 4.3 In vivo comparisons

**FIGURE 4:**
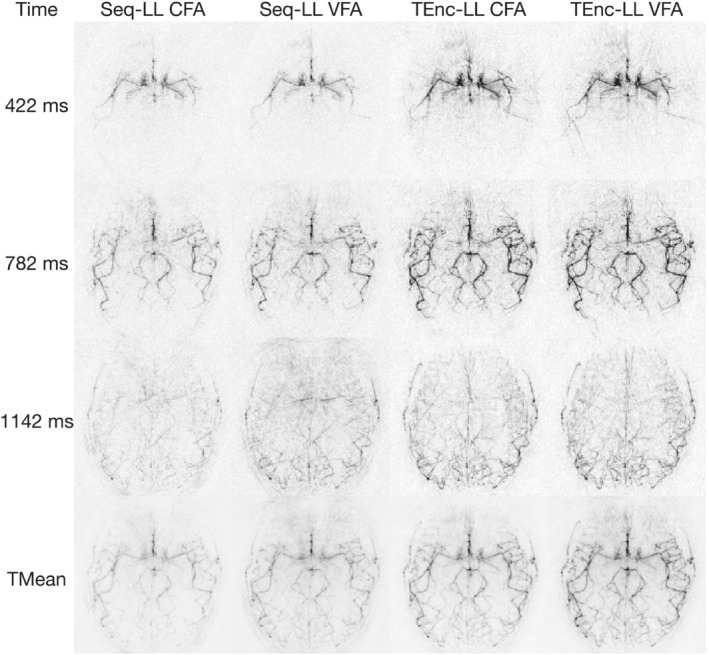
The fully sampled low‐resolution in vivo data for a single subject showing three selected PLDs and the temporal mean for each protocol. To aid in the visualization, 20 voxels around the edges of the FOV have been removed

For the low‐resolution comparison, Figure 4 shows three selected PLDs and the temporal mean for the Seq‐LL and TEnc‐LL CFA and VFA protocols for one volunteer. The signal gain from using TEnc‐LL versus Seq‐LL and VFA versus CFA is apparent at each PLD and in the temporal mean, where smaller vessels can be discerned. Supporting Information Figure S3 shows these data for all subjects at all PLDs and Supporting Information Figure S4 shows the same data as Figure 4 but each scan is windowed based on the expected ASL signal differences.

**FIGURE 5:**
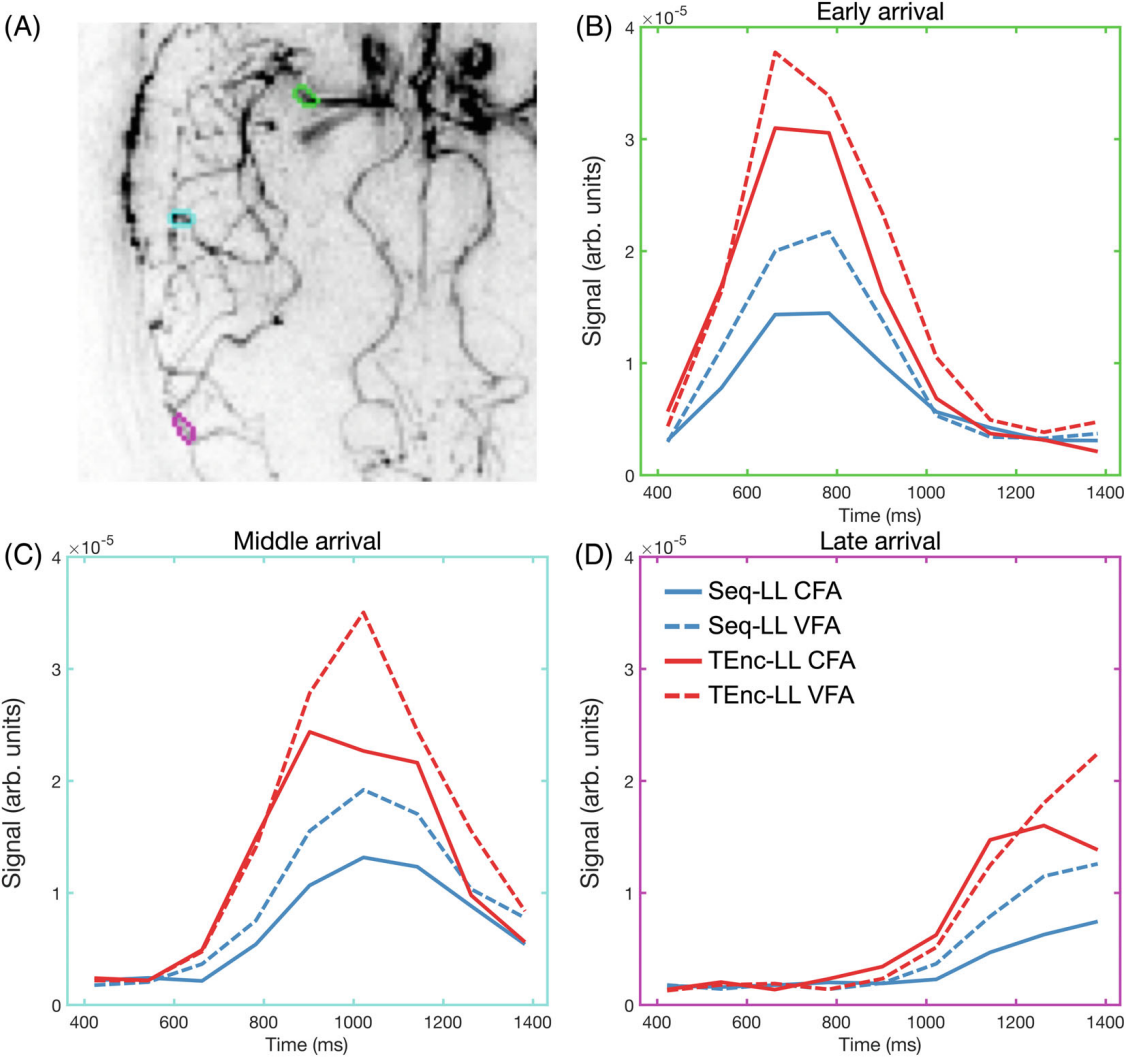
The in vivo signal time‐courses across PLDs in three vessel regions of interest (ROIs) in a single subject. A, The outline of the ROIs overlaid on the subject's temporal mean image. B–D, The ROIs show arteries with early (B), middle (C), and late (D) signal arrival. Each ROI contained eight voxels and were selected from the subject's vessel mask shown in Figure 2

Figure 5 shows the mean signal at each PLD within three vessel ROIs for one subject. An early, middle, and late‐arrival ROI are shown, demonstrating that the signal gains from using TEnc‐LL versus Seq‐LL and using VFAs versus CFAs is consistent across the vasculature. Although the bolus arrival time and apparent dispersion differ slightly, the TEnc‐LL CFA signal in Figure 5C has a signal that resembles the temporally down‐sampled simulations in Figure 3E, where the signal peak is lost due to the use of CFAs.

**FIGURE 6:**
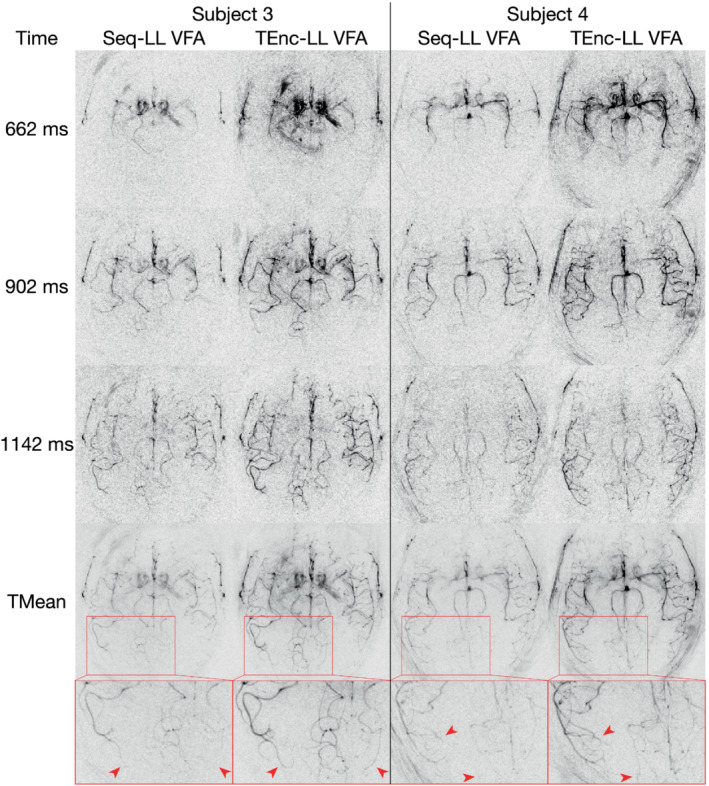
The high‐resolution in vivo data for two subjects showing three selected PLDs and the temporal mean for each protocol. To aid in the visualization, 40 voxels around the edges of the FOV have been removed. The zoomed sections of the temporal mean images illustrate the improved delineation of small distal vessels when using time encoding (red arrowheads)

The high‐resolution Seq‐LL and TEnc‐LL VFA data are shown in Figure 6 for two volunteers at three selected PLDs along with the temporal means. Similar signal gains can be seen at this resolution with a magnified section of the temporal means demonstrating improved small‐vessel depiction for the TEnc‐LL scan. Increased subtraction artifacts can be seen at some early PLDs in the TEnc‐LLVFA data due to the larger static tissue signal acquired than in the Seq‐LL protocol (see the simulations in Figure 9 for this effect). Supporting Information Figure S5 shows these data for all subjects at all PLDs, and Supporting Information Figure S6 shows these same data as Figure 6 but each scan is windowed based on the expected ASL signal differences.

#### 4.3.1 Signal‐to‐noise ratio quantification

**FIGURE 7:**
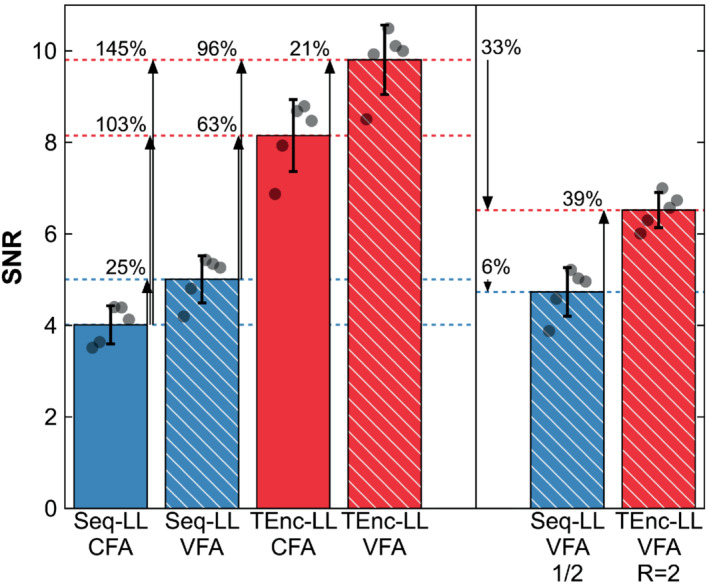
Quantified SNR for the low‐resolution data: two‐average/fully sampled (left) and one‐average/undersampled (right). The bar graphs and error bars show the mean and SD across subjects. The round markers show the SNR for each subject for each protocol. All differences in the fully sampled comparison were significant as was the difference in the undersampled comparison

The quantified SNR for the low‐resolution data is shown in Figure 7. The mean SNR was significantly higher in the time‐encoded scans compared with the Seq‐LL scans (103% increase for CFA and 96% increase for VFA, slightly larger improvements than predicted by simulation). The mean SNR improvement when using VFAs compared with CFAs was also significant (25% for Seq‐LL and 21% for TEnc‐LL, similar to the improvements predicted by simulation).

#### 4.3.2 Readout undersampling

**FIGURE 8:**
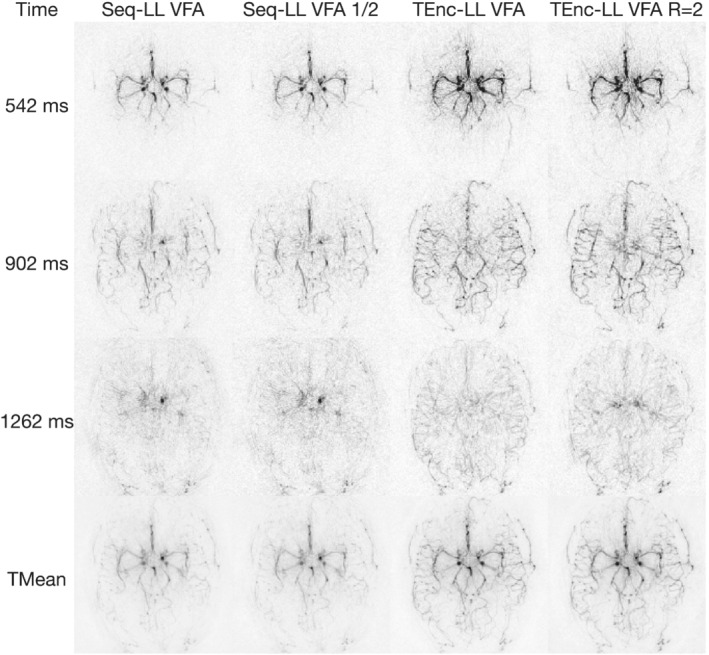
The low‐resolution in vivo data for a single subject comparing the two‐average/fully sampled data with the one‐average/undersampled Seq‐LL and TEnc‐LL data. Three selected PLDs and the temporal mean are shown for each protocol. To aid in the visualization, 20 voxels around the edges of the FOV have been removed

Figure 8 shows the one‐average Seq‐LL and prospectively angularly undersampled TEnc‐LL data at three selected PLDs and the temporal mean for one subject. Despite halving the number of averages for the Seq‐LL scan, the vessel visibility is very similar to the two‐average data. This is also the case for the TEnc‐LL data, despite the acquisition being angularly undersampled by a factor of ≈2. The vessels remain more visible for the undersampled TEnc‐LL data than the one‐average Seq‐LL data, demonstrating that the TEnc‐LL scan still provides a visibly obvious SNR advantage in a matched scan time compared with a standard Seq‐LL scan. Supporting Information Figure S7 shows these data for all subjects at all PLDs, and Supporting Information Figure S8 shows the same data as Figure 8 but each scan is windowed based on the expected ASL signal differences.

The quantitative SNR measures for this comparison are shown in Figure 7. While the Seq‐LL SNR only decreased by 6% when a single average was used, the TEnc‐LL SNR decreased by 33% when undersampled. Nonetheless, the TEnc‐LL SNR was still 39% higher than the Seq‐LL SNR.

#### 4.3.3 Optimized BGS

**FIGURE 9:**
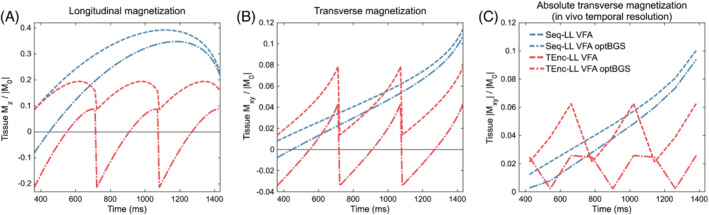
Simulations demonstrating the effect of optimizing the BGS (optBGS) null time (*t*_null,noRF_) on the normalized tissue magnetization for the VFA protocols (*T*_1t_ = 1.118 s). A, The longitudinal tissue magnetization immediately preceding each excitation pulse. B, The transverse magnetization immediately after each excitation pulse. C, The same as (B) but temporally downsampled to the temporal resolution used in vivo and after application of the magnitude operator, so as to be comparable with the in vivo data in Figure 10B. Temporal downsampling was performed by taking the mean signal across the 12 excitations in each LL frame. The time‐encoded signal for the three LL frames was repeated three times for visualization

The optimal BGS null times (*t*_null,noRF_) were −87 ms and −217 ms for the Seq‐LL and TEnc‐LL VFA protocols, respectively. These times corresponded to *T*_1t_ = 1.118 s being nulled about 85 ms and 187 ms after the first excitation, respectively, when accounting for the effect of the readout excitation pulses (Figure 9A). The null time of the Seq‐LL protocol was heavily constrained by the short ASL preparation and the long readout (the BGS inversion pulses could not be placed during the readout). The optimal null times were unchanged for *T*_1t_ > 0.5 s, so were robust to the exact *T*_1t_ value we used.

The simulated static tissue magnetization across the readouts for the Seq‐LL and TEnc‐LL VFA protocols, both with and without optimized BGS null times, are shown in Figure 9. The maximum absolute static tissue transverse magnetization has been greatly reduced for the TEnc‐LL protocol but has only been slightly reduced for the Seq‐LL protocol.

**FIGURE 10:**
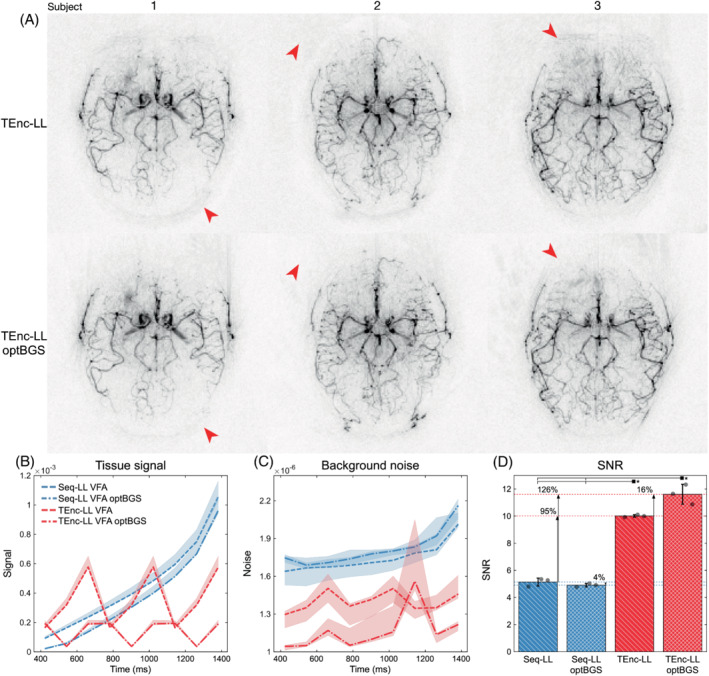
In vivo results demonstrating the impact of optimizing the BGS null time (*t*_null,noRF_) for the VFA protocols. A, The TEnc‐LL temporal means for each subject using unoptimized (100 ms) and optimized BGS null time, demonstrating an overall reduction in physiological noise. B, Subject‐wise in vivo static tissue signal (mean signal within brain mask across the four encoded images shown as median [line] and min‐max [error envelope] across subjects; the TEnc‐LL signal repeated 3 times for visualization). C, Subject‐wise in vivo background noise at each decoded timepoint (shown as median [line] and min‐max [error envelope] across subjects). D, Subject‐wise in vivo SNR for each protocol using the same vessel masks from Figure 2 (shown as mean [bar] and SD [error bars] across subjects; significant differences relative to TEnc‐LL and TEnc‐LL optBGS are highlighted with horizontal lines)

The in vivo tissue signal (mean signal within the brain mask) at each PLD for the three subjects in Figure 10C exhibits an excellent agreement with the simulations. Figure 10E demonstrates a reduction in the background noise for the TEnc‐LL protocol with the optimized null time, apart from at the seventh PLD. There appeared to be a small increase in background noise for the Seq‐LL protocol with optimized null time.

The in vivo SNR for the standard and optimized null times is shown in Figure 10D. The TEnc‐LL SNR increased by 16% (*p* = 0.056) and the Seq‐LL SNR decreased by 4% (*p* = 0.15), although neither difference was significant. The in vivo TEnc‐LL temporal means for each subject in Figure 10A highlight areas of reduced physiological noise near the back of the skull, the eyes, and aliased outside the head. Supporting Information Figure S9 shows these data at each PLD.

## 5 DISCUSSION

We have demonstrated that the use of time‐encoded PCASL enables the number of excitations in the LL readout to be reduced, which facilitates the use of larger flip angles. This approach can approximately double the mean angiographic SNR compared with a time‐matched conventional protocol, Seq‐LL. A VFA scheme that maintains a constant ASL signal throughout the readout results in a smoother signal time‐course and increases SNR by a further 21% (145% higher than Seq‐LL with CFAs).

By angularly undersampling the TEnc‐LL acquisition by a factor of ≈2, the scan time was reduced to a similar duration to that of a single average of the Seq‐LL protocol. Despite this large reduction in k‐space sampling, most of the arteries were still clearly visible and there was still a large SNR benefit over the Seq‐LL protocol.

Finally, we demonstrated that optimization of the BGS null time can lead to improved static tissue suppression during the entire readout, which can reduce the physiological noise present in the angiograms. This was of particular benefit to the TEnc‐LL protocol because the long PCASL preparation relative to the readout duration meant that the tissue magnetization could be nulled close to the optimal time and a smaller acquired tissue magnetization could be maintained throughout the shorter readout. The short PCASL preparation and long readout duration of the Seq‐LL protocol heavily constrained the possible BGS timings and meant that the achieved level of BGS was only slightly better than the original BGS.

### 5.1 Label duration

We chose the relatively short LD of 360 ms in order to match the timings of the Seq‐LL and TEnc‐LL protocols. A benefit of short LDs is that arterial inflow can be visualized^9^; if a long LD were used, only the outflow could be visualized. While “inflow subtraction”^41^, ^42^ (where each frame is subtracted from the first frame) can be used to visualize inflow with longer LDs, it increases noise and assumes that all vessels are filled with labeled blood at the start of the readout. Short LDs also reduce scan time; for example, a LD of 1 s would increase the Seq‐LL protocol scan time from 2:30 min to 3:34 min (low resolution) and 4:35 min to 6:33 min (high resolution). Nonetheless, a long LD could improve the depiction of distal vessels because labeled blood may have reached them before starting to be attenuated by the LL readout. Furthermore, under severe dispersion, the peak signal can be reduced when using a short LD due to the temporal smearing of the bolus, while a long LD would still reach a maximum plateau.^43^, ^44^

### 5.2 Protocol optimization

The protocol timings were chosen to directly compare the SNR of matched Seq‐LL and TEnc‐LL protocols, similar to the perfusion comparison of Dai et al.^16^ In addition to using dynamic angiograms for qualitative assessment of arterial supply, they can provide quantitative measurements of blood volume, transit times, and dispersion parameters when fit with an appropriate kinetic model.^24^, ^45^ For this purpose, each protocol may perform best when using a different set of protocol timings, which could be found using a Cramér‐Rao lower bound framework to minimize measurement error.^46^ This approach could provide insight into the relative merits of each protocol type without constraining the protocol timings to being identical, in a similar way to recent work in quantitative perfusion imaging.^18^

### 5.3 Flip‐angle optimization

The VFA scheme increased the acquired ASL signal compared with CFAs in simulations and in vivo by more effectively utilizing the available magnetization. Several downsides of this flip‐angle scheme include higher specific absorption rate and increased tissue subtraction artifacts at some PLDs when the BGS is not optimized for the entire readout. While the signal attenuation during the CFA readout could be adjusted for in postprocessing to remove the discontinuities in the decoded TEnc‐LL data, this would also scale the noise, meaning that the SNR would remain unchanged and lower than the VFA data.

For flip‐angle optimization, the minimum signal during the readout was maximized (max‐min), which was equivalent to maximizing the signal at the last excitation for the schemes used here. We chose this aim to improve the visibility of small distal vessels,^47^ which are most likely to be visualized at the end of the readout when the ASL bolus has traveled furthest down the arterial tree.

An alternative aim for the CFAs could have been to maximize the mean (max‐mean) signal across the readout. This would have, theoretically, resulted in a 15% and 14% higher mean signal than max‐min for Seq‐LL and TEnc‐LL, respectively. However, the signal at the last excitation would have been 25% lower for both protocols compared with max‐min, severely compromising SNR at the end of the readout. Additionally, the mean signal for the max‐mean CFA case would still have been 9% and 6% lower than for the VFA readout for Seq‐LL and TEnc‐LL protocols, respectively. This comparison is shown in Supporting Information Figure S10.

An alternative VFA formulation to further improve distal vessel visibility could aim to increase the acquired ASL signal throughout the readout, increasing the signal as the tracer travels downstream into smaller vessels. However, this would result in a gradual brightening of the label within a given voxel during the readout and would also cause the ASL signal to oscillate for the TEnc‐LL protocol similar to CFAs.

The flip‐angle optimizations assumed that all ASL signal experienced every readout pulse. However, this is unlikely to have been the case with the experimental setup used in this work. For example, one volunteer had a 53‐mm gap between the imaging volume and the labeling plane. Assuming a constant blood velocity of 40 cm/s and straight vessels perpendicular to the labeling plane and imaging volume, it would take 132.5 ms for labeled blood to reach the image volume—much longer than the 2‐ms gap between the end of labeling and the start of the readout. While this likely only has a small visual effect, the resulting signal variations would need to be taken into account for quantification of arterial blood volume.^24^

### 5.4 Signal‐to‐noise ratio quantification

The background‐noise ROIs will incorporate both thermal noise and signal originating from the subject due to the imperfect point spread function, trajectory errors, gridding process, and flow artifacts. When the radial acquisition is angularly undersampled, these background noise ROIs will also include increased aliased signal. However, this may not greatly affect visualization of the arteries, because a circular region with diameter $\frac{\mathrm{FOV}}{2}$ around each signal source will only contain a small amount of aliased signal.^48^ If most of the arteries with significant signal are within this distance of each other, the undersampling artifacts will not greatly affect vessel visibility. The SNR calculation for the undersampled TEnc‐LL data, therefore, will likely be an underestimate of the effective SNR in the local neighborhood of the vessels, and its effective advantage over the one‐average Seq‐LL data is expected to be larger than that seen in Figure 8.

### 5.5 Optimized BGS

The BGS null time for each protocol was optimized to minimize the maximum absolute static tissue signal acquired during the readout. After optimization, the in vivo static tissue was better suppressed across the entire readout. This led to a general reduction in the background noise for the TEnc‐LL protocol, but a small increase in noise for the Seq‐LL protocol. Additionally, the noise at the seventh PLD of the time‐encoded protocol was increased for all three subjects. This noise did not appear to be due to motion, because it appeared as a general increase in noise across the entire FOV and it was consistent across subjects (see Supporting Information Figure S9). This consistency suggests that it may be due to an interaction between the BGS inversion pulse timings and the encoding pattern of the first time‐encoded block, although further work is required to investigate this.

Overall, the reduced background noise for the BGS‐optimized TEnc‐LL protocol led to a further 16% increase in SNR on average compared with nulling static tissue before the readout, although this improvement was not significant. Only three volunteers were scanned, so further validation is required to be confident in the size of the SNR improvements. Nonetheless, it is widely accepted that reducing static tissue signal reduces physiological noise,^49^, ^50^ so despite the limited in vivo demonstration here, we would expect this approach to be generally beneficial.

The BGS scheme could be made to be more flexible by allowing the inversion pulses to be placed during the readout, achieving improved background tissue suppression on average. Alternatively, additional inversion pulses could be placed during the readout to maintain minimal background signal throughout the entire readout,^51^, ^52^ although at the cost of increased ASL signal attenuation at later timepoints due to imperfect inversions.

### 5.6 Time encoding

A potential drawback of using time encoding compared with conventional ASL is that it is more sensitive to any signal changes that occur across the scan that are not due to labeled blood (eg, subject motion, pulsatility, B_0_ drift), because more than two images are used to generate the difference images.^53^ This typically means the required data are acquired over a longer period of time, during which such effects could occur. Similarly, it is possible that time encoding is more sensitive to pathological flow conditions, such as in aneurysms and arteriovenous malformations, although this would need to be explored in future work. Sensitivity to subject motion in general is reduced when using effective BGS^49^ and radial readouts,^54^ but could be further reduced by using retrospective self‐navigated motion correction with golden angle radial ordering.^55^, ^56^, ^57^

Rather than combining time encoding with multiple readout frames to generate the temporal information, time encoding could be the sole source. For example, an 8 × 7 Hadamard encoding matrix with a single readout frame would enable larger flip angles while still yielding seven PLDs.^9^ However, to maintain a similar range of PLDs to the protocols in this study, the LD would need to be very short (160 ms for PLDs between 62 ms and 1022 ms), potentially leading to a decreased peak signal due to dispersion.^44^ A larger encoding matrix also increases sensitivity to subtraction artifacts due to motion. Additionally, to avoid increasing the scan time compared with the single‐average Seq‐LL protocol, the readout would need to be undersampled by a factor of 4 rather than 2, further increasing aliasing artifacts.

While we kept the LD constant for each time‐encoded block for this work, there is no requirement to do so in general.^44^ Increasing the LD for the first block will not affect the PLDs or the visualization of inflow, and therefore could be used to further fill the distal vasculature for the longest PLD.^9^ However, this approach will increase scan time.

### 5.7 Pseudo‐continuous ASL versus pulsed ASL

Pulsed ASL (PASL) can also be used for ASL angiography.^6^ Because the PASL labeling period is extremely short (≈10 ms), imaging can begin almost immediately and visualize the entire inflow of blood. However, as previously mentioned, most of the inflow is also visualized with the PCASL protocols used in this study due to the short LD. Another benefit of PASL is that the labeling efficiency is higher than PCASL (≈98%^58^ vs ≈85%^59^), but this neglects that the entire PASL bolus is created simultaneously and upstream labeled blood has further to travel, resulting in a lower effective mean labeling efficiency, *α*, across the bolus (≈79% for the entire bolus [LD = 800 ms^58^] or ≈90% for the bolus size used in this study [LD = 360 ms], where $\alpha =\frac{1}{\mathrm{LD}}\int_{0}^{\mathrm{LD}}e^{-\frac{t}{T_{1b}}}\cdot \mathrm{dt}$ and *T*_1b_ = 1.65 s). Nonetheless, distal vessel visibility will still benefit from the higher labeling efficiency at the front of the PASL bolus.

Most importantly, however, PASL cannot practically be used with time encoding, so all of the temporal information would need to be generated from the LL readout, requiring low flip angles, which lead to low SNR. Given the large SNR improvements achieved when using time encoding, we suspect that the TEnc‐LL protocol could outperform an equivalent PASL protocol, although this would need to be confirmed in future studies.

The time‐encoded PCASL protocol presented here could also be combined with PASL to extend the inflow of labeled blood during the readout.^52^, ^60^, ^61^ Finally, PCASL has the benefit of being able to selectively label arteries,^43^, ^62^, ^63^, ^64^ which can be difficult with PASL in practice,^27^ particularly when selecting distal vessels.

### 5.8 Cardiac triggering

We did not use cardiac triggering in this study, but it may lead to a sharper bolus profile and increased peak signal,^44^ improving data quality for physiological modeling and potentially improving distal vessel depiction. However, because cardiac triggering has a relatively small effect on the total volume of labeled blood,^44^, ^65^ the results of the quantitative SNR comparison (Figure 7) are not expected to change because the average SNR across the arterial tree was calculated. Additionally, cardiac pulsatility is expected to affect both protocols similarly.^44^

### 5.9 Readout

We demonstrated the SNR benefits of time encoding using a 2D radial SPGR readout to enable all scans to be performed within a single session for each subject, but similar SNR benefits can also be expected with other readouts.

Extending this approach to 3D radial is relatively simple, for example by using stack‐of‐stars,^66^ in which the third dimension is phase‐encoded, or Koosh‐Ball trajectories,^67^, ^68^, ^69^, ^70^ where the radial spokes are rotated around two axes. Identical protocol timings and VFAs to those used in this study can still be used, but many more spokes will need to be acquired during additional TRs in order to fill the 3D k‐space, leading to very long scan durations. However, due to the inherently sparse nature of 3D angiograms, compressed sensing^71^ can be used to greatly reduce scan durations to clinically feasible times with only minor reductions in image quality.^20^, ^72^, ^73^, ^74^, ^75^

Balanced SSFP^76^ provides greater SNR than SPGR because residual transverse magnetization is re‐used in later TRs rather than being spoiled after each excitation. Time encoding would enable even larger flip angles than are currently possible with ASL angiography using balanced SSFP.^42^ However, there will still be signal attenuation during a balanced SSFP readout using a CFA, due to T_2_/T_2_* relaxation leading to discontinuities in the decoded time‐encoded data, although this could be resolved with a balanced SSFP VFA approach.^77^, ^78^ Nonetheless, it is more challenging to extend balanced SSFP to whole‐brain 3D readouts at 3 T,^42^ which we aim to do in future work, due to the technique's sensitivity to off‐resonance and the difficulty in achieving a sufficiently good shim across the entire brain, so it is more commonly used at 1.5 T.^52^, ^60^

## 6 CONCLUSIONS

We have demonstrated that a time‐encoded preparation can be beneficial for dynamic PCASL angiography by allowing some of the temporal information to be generated from the ASL preparation. This enabled the use of larger flip angles during a shorter LL readout, which led to an SNR improvement of 103%. Furthermore, a VFA formula was proposed that removed the signal discontinuities present in the time‐encoded data when using CFAs and led to a further SNR increase of 21% (145% relative to the Seq‐LL CFA protocol). By optimizing the BGS to maintain a low background tissue signal across the readout, the SNR can potentially be increased by a further 16%.

## FUNDING INFORMATION

TWO and JGW were supported by a Sir Henry Dale Fellowship jointly funded by the Wellcome Trust and the Royal Society (220204/Z/20/Z). JGW, SSS, and MAC were supported by funding from the Engineering and Physical Sciences Research Council (EP/L016052/1 and EP/P012361/1). The Wellcome Centre for Integrative Neuroimaging is supported by core funding from the Wellcome Trust (203139/Z/16/Z). For the purpose of open access, the author has applied a CC BY public copyright license to any author‐accepted manuscript version arising from this submission.

## Supporting information


**FIGURE S1.** A,B, Comparison of the flip angles (A) and transverse arterial spin labeling (ASL) magnetization (B) for the variable flip angle (VFA) scheme when using either a maximum flip angle of 30° or 90°. The decrease in signal after reducing the maximum flip angle from 90° to 30° for the sequential Look‐Locker (Seq‐LL) and time‐encoded Look‐Locker (TEnc‐LL) protocols was 1% and 4%, respectively. As in Figure 3B, a constant supply of ASL signal and zero arrival time was assumed
**FIGURE S2.** Comparing data reconstructed without and with the phase correction described section 3. The use of phase correction (PC) helped reduce subtraction errors, most notably at the back of the brain at later timepoints when the static tissue signal was larger
**FIGURE S3.** Animation showing the fully sampled low‐resolution data for all five subjects at all nine PLDs. Each row shows a different subject. The columns are (left to right): Seq‐LL constant flip angle (CFA), Seq‐LL VFA, TEnc‐LL CFA, and TEnc‐LL VFA
**FIGURE S4.** The same data are shown as Figure 4 but with the data from each scan (Seq‐LL CFA, Seq‐LL VFA, TEnc‐LL CFA, and TEnc‐LL VFA) individually windowed based on the expected mean ASL signal differences from the simulations in Figure 3B
**FIGURE S5.** Animation showing the high‐resolution data for all four subjects at all nine PLDs. Each row shows a different subject. The columns are (left to right): Seq‐LL VFA and TEnc‐LL VFA
**FIGURE S6.** The same data as Figure 6 but with the data from each scan (Seq‐LL CFA and TEnc‐LL VFA) individually windowed based on the expected mean ASL signal differences from the simulations in Figure 3B
**FIGURE S7.** Animation showing the one‐average/undersampled low‐resolution data for all five subjects at all nine PLDs. Each row shows a different subject. The columns are (left to right): one‐average Seq‐LL VFA and two‐times undersampled TEnc‐LL VFA
**FIGURE S8.** The same data as Figure 8 but with the data from each scan (Seq‐LL CFA and TEnc‐LL VFA) individually windowed based on the expected mean ASL signal differences from the simulations in Figure 3B
**FIGURE S9.** Animation showing the original background suppression (BGS; left column) and optimized null time BGS (right column) case for the fully sampled low‐resolution TEnc‐LL VFA data for all three subjects at all nine PLDs. Each row shows a different subject. The higher level of background noise in the optimized BGS data at the seventh PLD can be seen consistently for each subject and appears as an increased noise level across the whole FOV
**FIGURE S10.** A, The optimized flip angles for the max‐min CFA, max‐mean CFA, and VFA schemes for the Seq‐LL and TEnc‐LL protocols. B, The simulated acquired ASL signal for each set of flip angles assuming a constant supply of ASL signal and zero arrival time. The CFAs were Seq‐LL max‐min = 5.5°, Seq‐LL max‐mean = 8.7°, TEnc‐LL max‐min = 9.6°, and TEnc‐LL max‐mean = 15.1°

## Data Availability

In support of Magnetic Resonance in Medicine's reproducible research goal, the raw k‐space data and *MATLAB* code used for reconstruction, simulations, data analysis, and figure generation for this manuscript are available at https://doi.org/10.5281/zenodo.6791097.
