# Supplementary material for: Time‐encoded pseudo‐continuous arterial spin labeling: Increasing SNR in ASL dynamic angiography
Source: Magn Reson Med. 2022 Oct 18;89(4):1323–41. doi: 10.1002/mrm.29491 (PMC10091734; doi:10.1002/mrm.29491)
Supplement: Supplementary file 1 — FIGURE S1. A,B, Comparison of the flip angles (A) and transverse arterial spin labeling (ASL) magnetization (B) for the variable flip angle (VFA) scheme when using either a maximum flip angle of 30° or 90°. The decrease in signal after reducing the maximum flip angle from 90° to 30° for the sequential Look‐Locker (Seq‐LL) and time‐encoded Look‐Locker (TEnc‐LL) protocols was 1% and 4%, respectively. As in Figure 3B, a constant supply of ASL signal and zero arrival time was assumed FIGURE S2. Comparing data reconstructed without and with the phase correction described section 3. The use of phase correction (PC) helped reduce subtraction errors, most notably at the back of the brain at later timepoints when the static tissue signal was larger FIGURE S3. Animation showing the fully sampled low‐resolution data for all five subjects at all nine PLDs. Each row shows a different subject. The columns are (left to right): Seq‐LL constant flip angle (CFA), Seq‐LL VFA, TEnc‐LL CFA, and TEnc‐LL VFA FIGURE S4. The same data are shown as Figure 4 but with the data from each scan (Seq‐LL CFA, Seq‐LL VFA, TEnc‐LL CFA, and TEnc‐LL VFA) individually windowed based on the expected mean ASL signal differences from the simulations in Figure 3B FIGURE S5. Animation showing the high‐resolution data for all four subjects at all nine PLDs. Each row shows a different subject. The columns are (left to right): Seq‐LL VFA and TEnc‐LL VFA FIGURE S6. The same data as Figure 6 but with the data from each scan (Seq‐LL CFA and TEnc‐LL VFA) individually windowed based on the expected mean ASL signal differences from the simulations in Figure 3B FIGURE S7. Animation showing the one‐average/undersampled low‐resolution data for all five subjects at all nine PLDs. Each row shows a different subject. The columns are (left to right): one‐average Seq‐LL VFA and two‐times undersampled TEnc‐LL VFA FIGURE S8. The same data as Figure 8 but with the data from each scan (Seq‐LL CFA and TEnc‐LL VFA) individually wi [file MRM-89-1323-s001.docx]

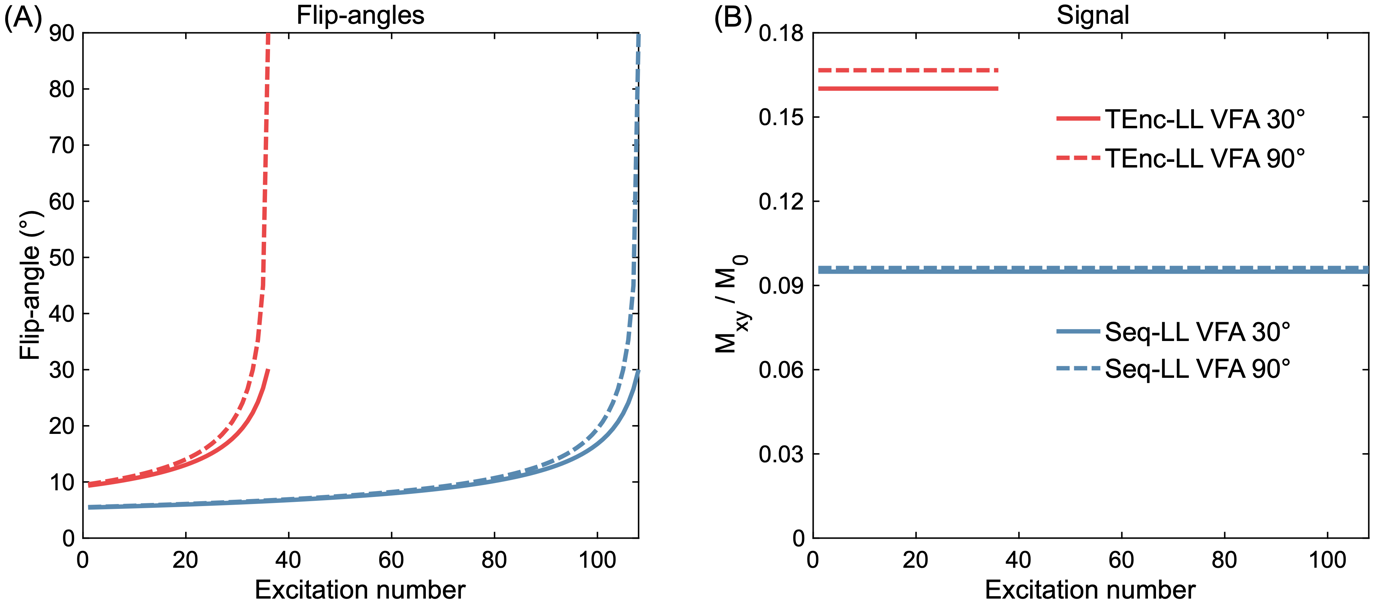


Supporting Information Figure S1: Comparison of the flip angles (A) and simulated acquired ASL signal (B) for the VFA scheme when using either a maximum flip angle of 30° or 90°. The decrease in the simulated acquired signal after reducing the maximum flip angle from 90° to 30° for the Seq-LL and TEnc-LL protocols was 1% and 4%, respectively. As in Figure3B, a constant supply of ASL signal and zero arrival time was assumed.


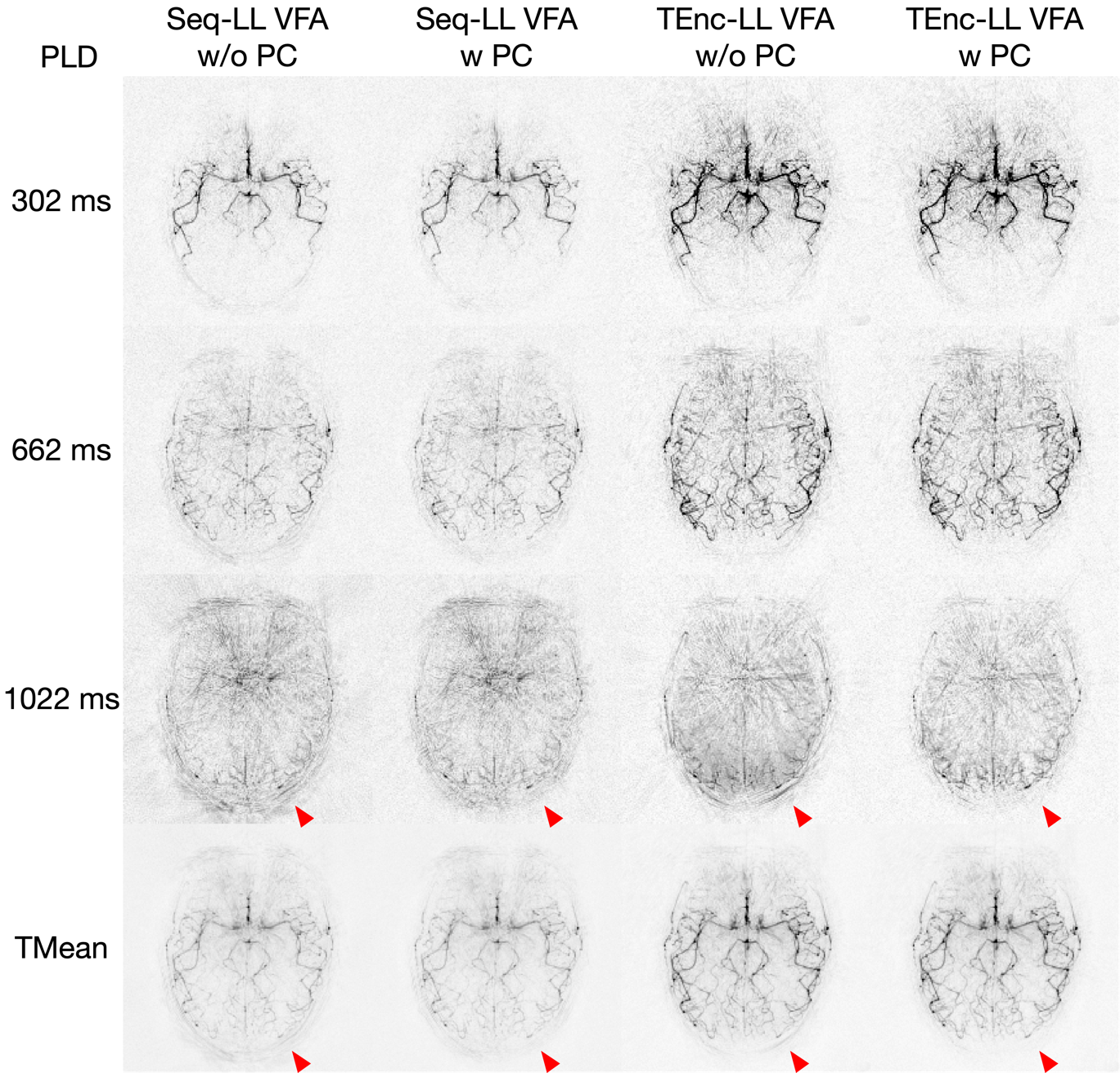


Supporting Information Figure S2: Comparing data reconstructed without and with the phase correction described in the methods section. The use of phase correction helped reduce subtraction errors, most notably at the back of the brain at later time points, when the static tissue signal was larger.


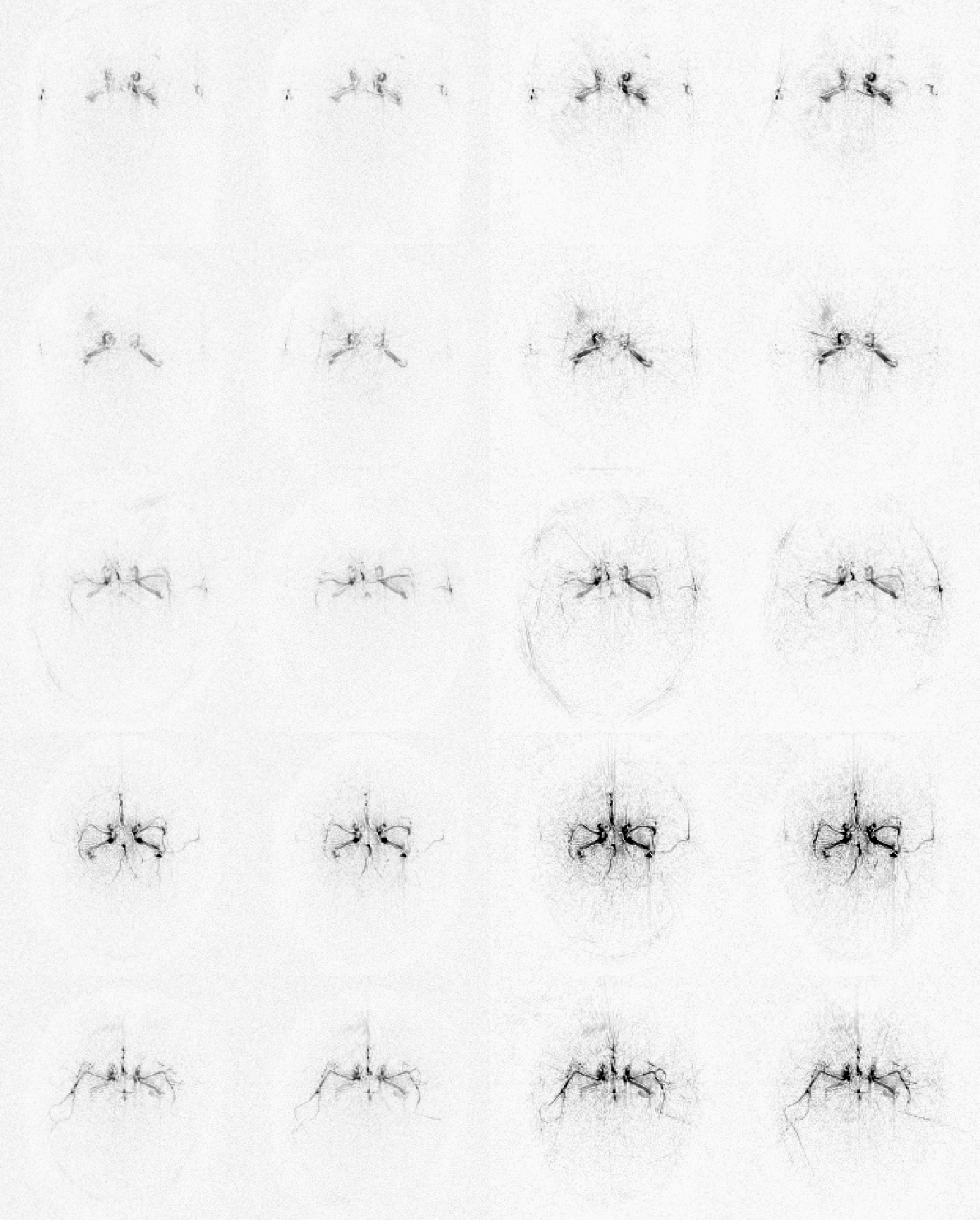


Supporting Information Figure S3: Animation showing the fully-sampled low-resolution data for all 5 subjects at all 9 PLDs. Each row shows a different subject. The columns are (left to right): Seq-LL CFA, Seq-LL VFA, TEnc-LL CFA, and TEnc-LL VFA.


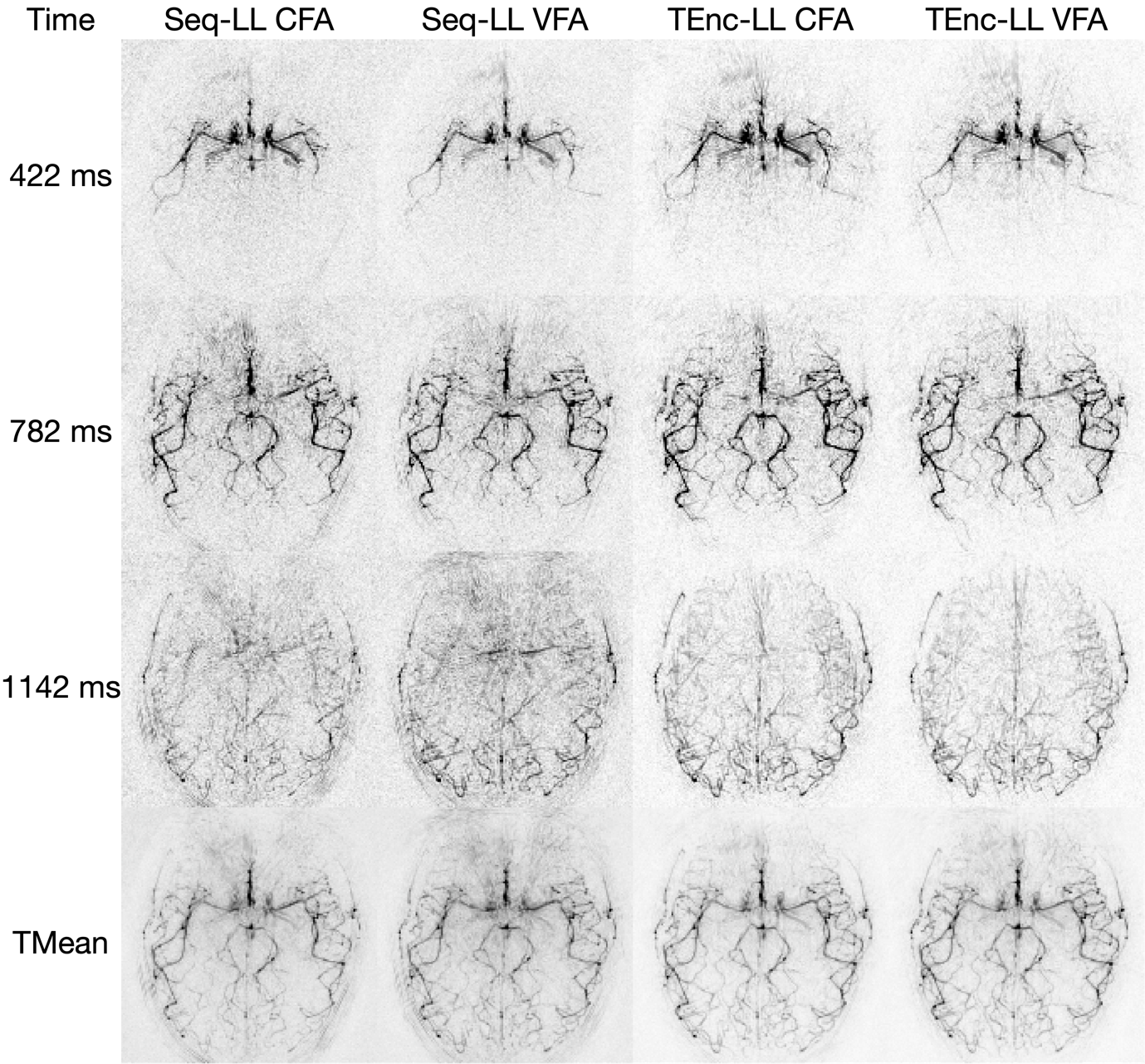


Supporting Information Figure S4: The same data as Figure 4 but with the data from each scan (Seq-LL CFA, Seq-LL VFA, TEnc-LL CFA, and TEnc-LL VFA) individually windowed based on the expected mean ASL signal differences from the simulations in Figure 3B.


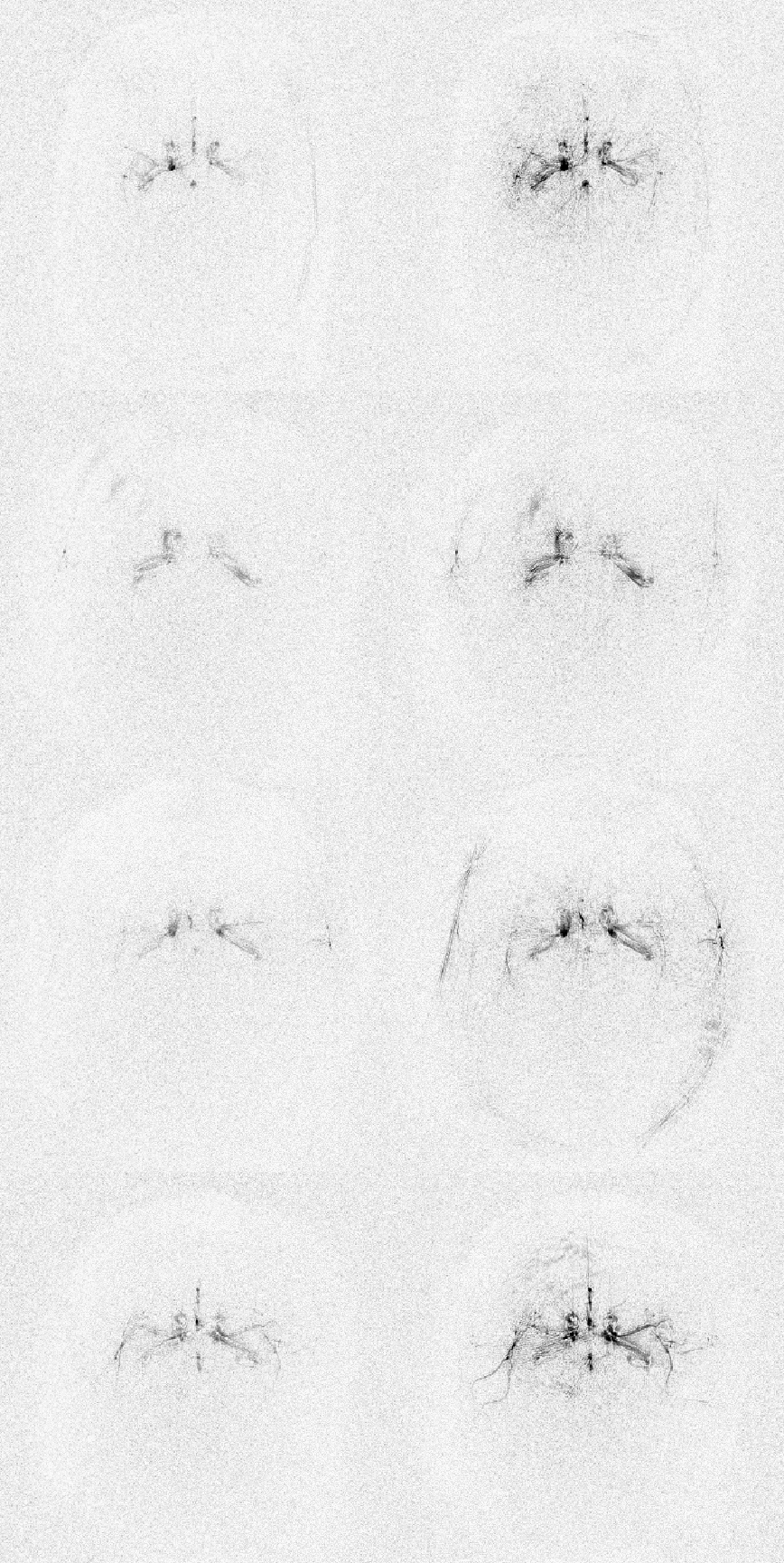


Supporting Information Figure S5: Animation showing the high-resolution data for all 4 subjects at all 9 PLDs. Each row shows a different subject. The columns are (left to right): Seq-LL VFA and TEnc-LL VFA.


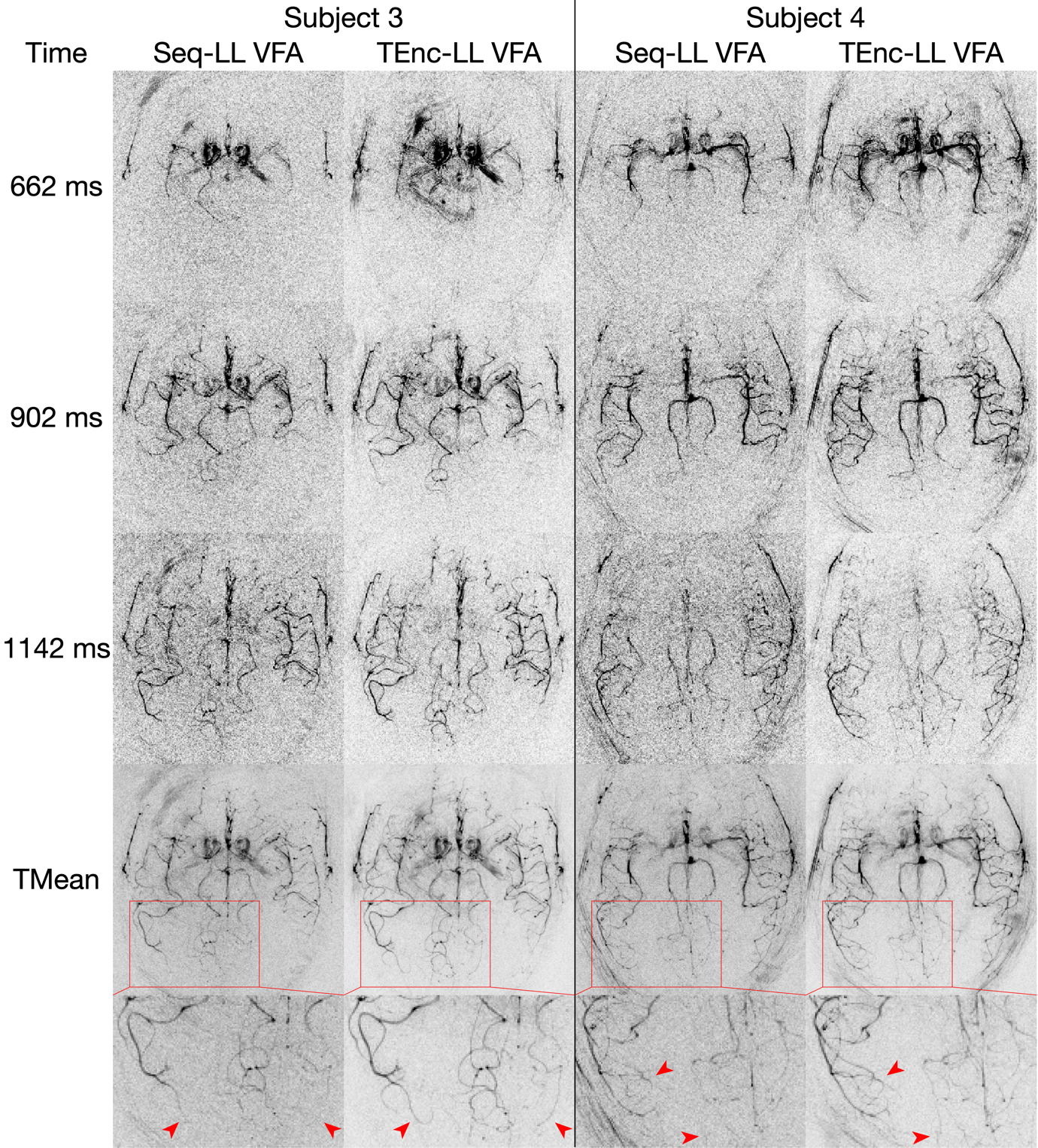


Supporting Information Figure S6: The same data as Figure 6 but with the data from each scan (Seq-LL CFA and TEnc-LL VFA) individually windowed based on the expected mean ASL signal differences from the simulations in Figure 3B.


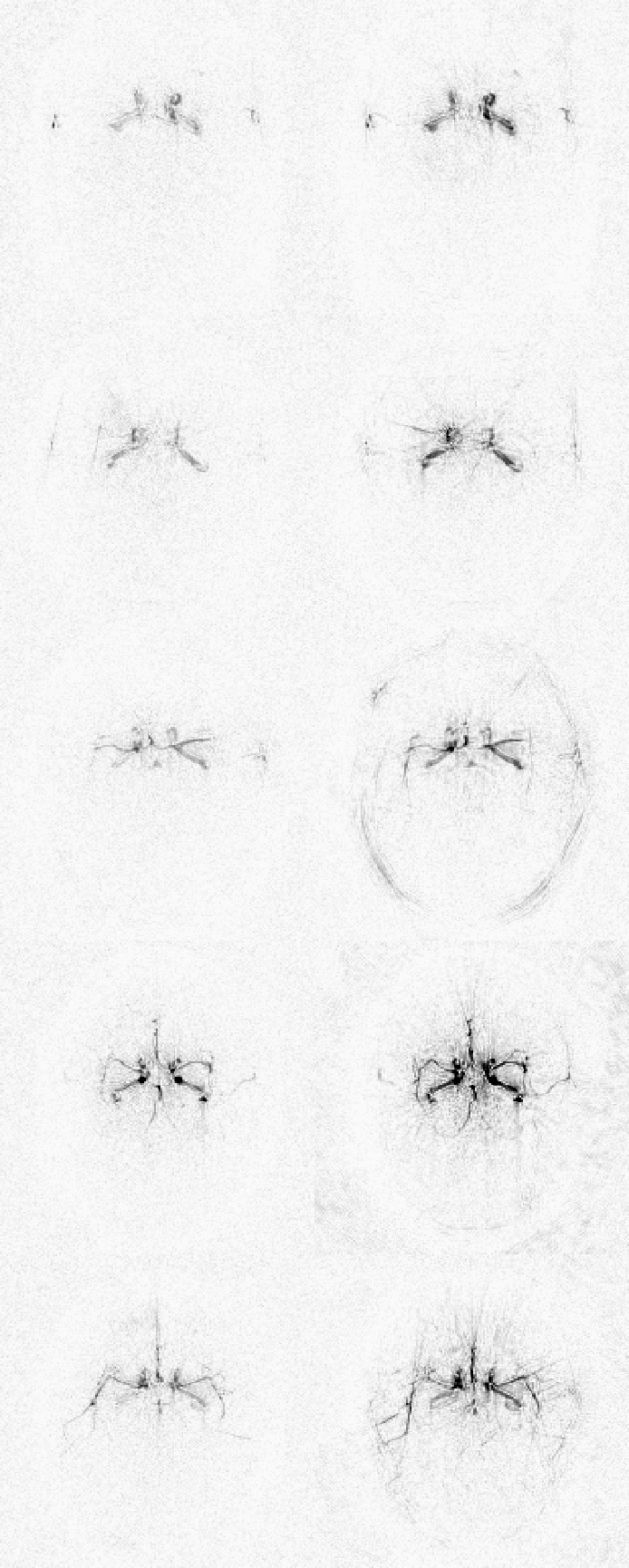


Supporting Information Figure S7: Animation showing the 1-average/under-sampled low-resolution data for all 5 subjects at all 9 PLDs. Each row shows a different subject. The columns are (left to right): 1-average Seq-LL VFA and 2x under-sampled TEnc-LL VFA.


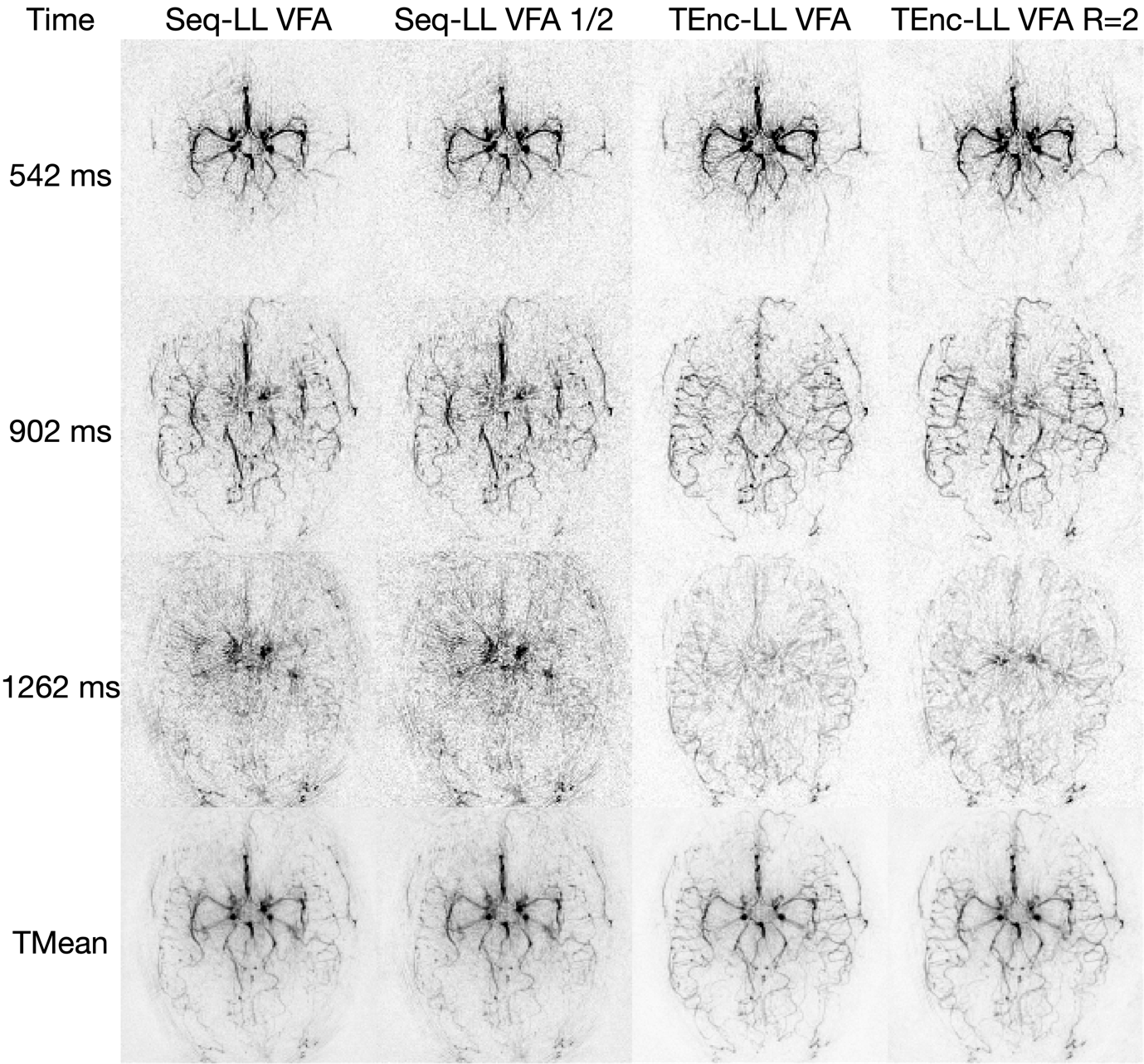


Supporting Information Figure S8: The same data as Figure 8 but with the data from each scan (Seq-LL CFA and TEnc-LL VFA) individually windowed based on the expected mean ASL signal differences from the simulations in Figure 3B.


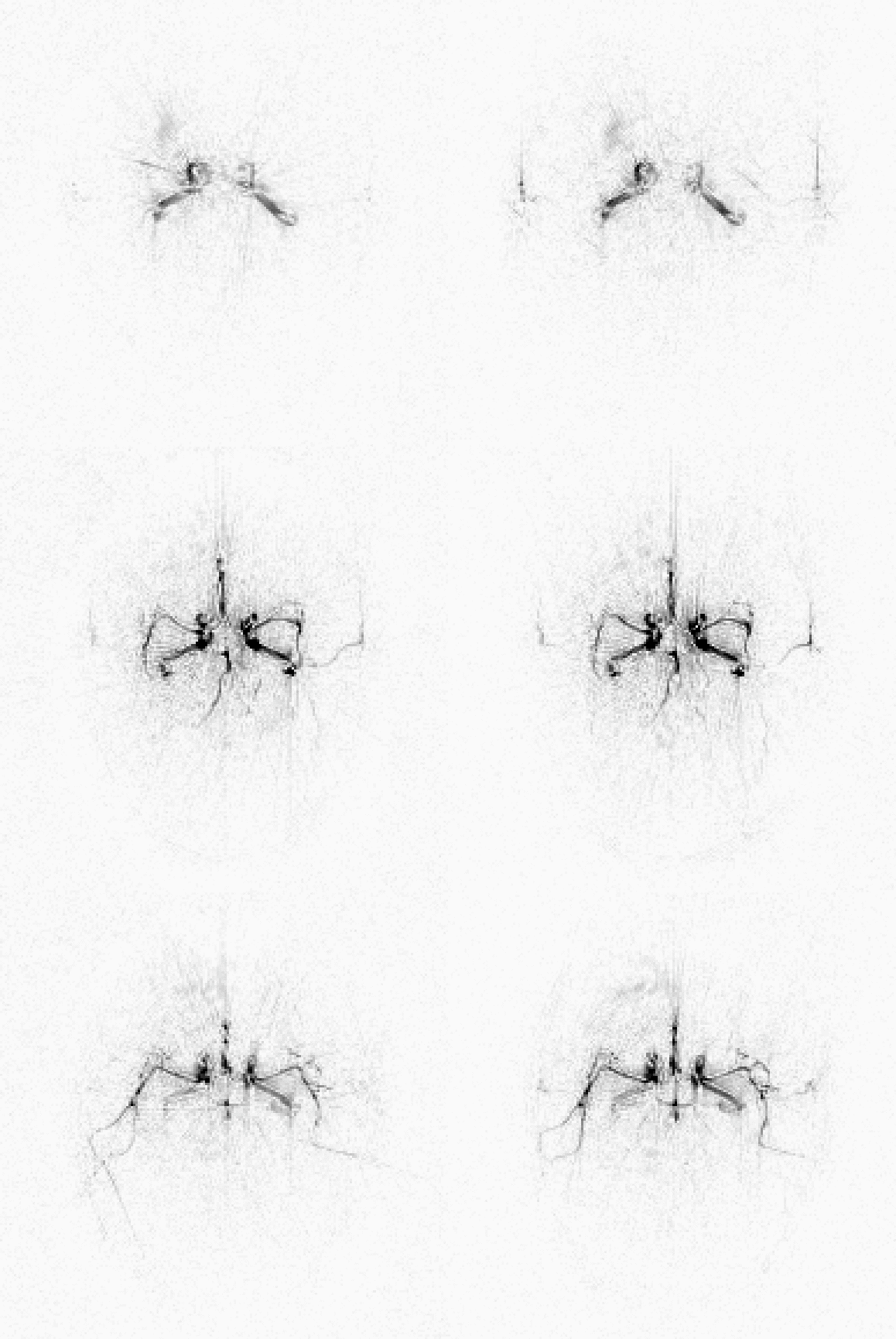


Supporting Information Figure S9: Animation showing the original BGS (left column) and optimized null time BGS (right column) case for the fully-sampled low-resolution TEnc-LL VFA data for all 3 subjects at all 9 PLDs. Each row shows a different subject. The higher level of background noise in the optimized BGS data at the 7th PLD can be consistently seen for each subject and appears as an increased noise level across the whole FOV.


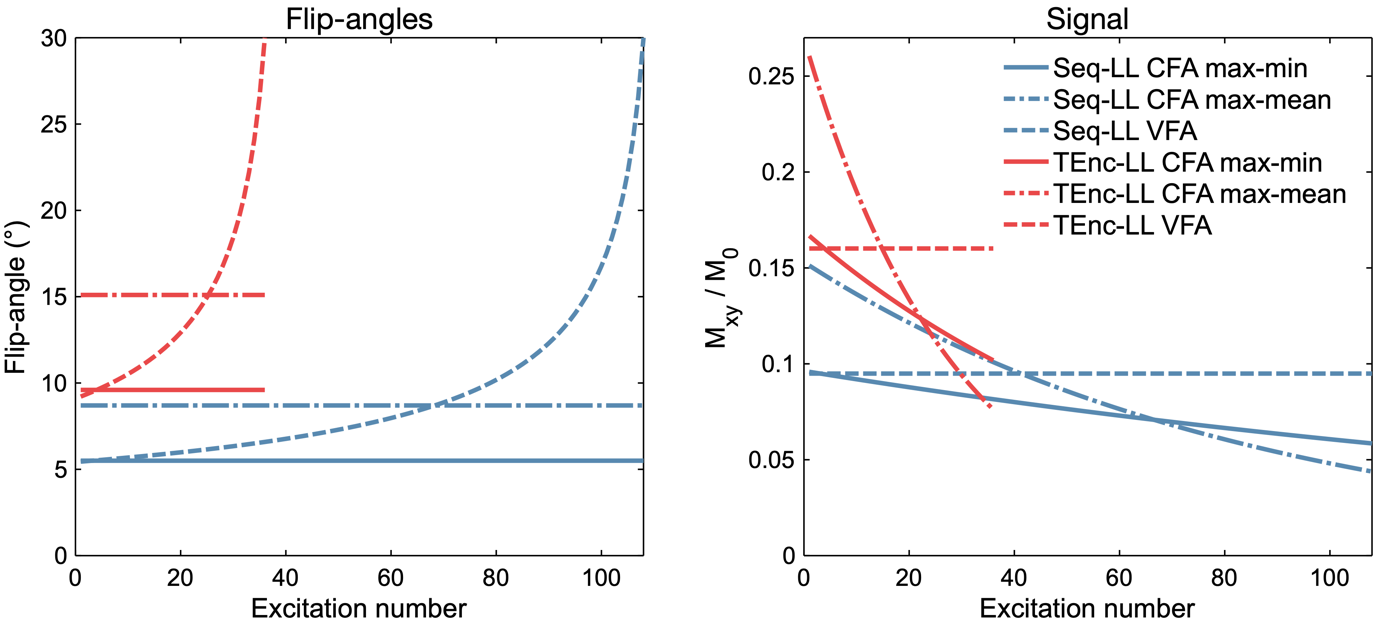


Supporting Information Figure S10: (A) The optimized flip angles for the max-min CFA, max-mean CFA and VFA schemes for the Seq-LL and TEnc-LL protocols. (B) The simulated acquired ASL signal for each set of flip angles assuming a constant supply of ASL signal and zero arrival time. The CFAs were Seq-LL max-min = 5.5°, Seq-LL max-mean = 8.7°, TEnc-LL max-min = 9.6°, TEnc-LL max-mean = 15.1°.
